# Predicting and analyzing the COVID-19 epidemic in China: Based on SEIRD, LSTM and GWR models

**DOI:** 10.1371/journal.pone.0238280

**Published:** 2020-08-27

**Authors:** Fenglin Liu, Jie Wang, Jiawen Liu, Yue Li, Dagong Liu, Junliang Tong, Zhuoqun Li, Dan Yu, Yifan Fan, Xiaohui Bi, Xueting Zhang, Steven Mo

**Affiliations:** Taikang Pension & Insurance Co., Ltd., Beijing, China; Faculty of Science, Ain Shams University (ASU), EGYPT

## Abstract

In December 2019, the novel coronavirus pneumonia (COVID-19) occurred in Wuhan, Hubei Province, China. The epidemic quickly broke out and spread throughout the country. Now it becomes a pandemic that affects the whole world. In this study, three models were used to fit and predict the epidemic situation in China: a modified SEIRD (Susceptible-Exposed-Infected-Recovered-Dead) dynamic model, a neural network method LSTM (Long Short-Term Memory), and a GWR (Geographically Weighted Regression) model reflecting spatial heterogeneity. Overall, all the three models performed well with great accuracy. The dynamic SEIRD prediction APE (absolute percent error) of China had been ≤ 1.0% since Mid-February. The LSTM model showed comparable accuracy. The GWR model took into account the influence of geographical differences, with R^2^ = 99.98% in fitting and 97.95% in prediction. Wilcoxon test showed that none of the three models outperformed the other two at the significance level of 0.05. The parametric analysis of the infectious rate and recovery rate demonstrated that China's national policies had effectively slowed down the spread of the epidemic. Furthermore, the models in this study provided a wide range of implications for other countries to predict the short-term and long-term trend of COVID-19, and to evaluate the intensity and effect of their interventions.

## Introduction

Novel coronavirus pneumonia (coronavirus disease 2019, COVID-19) break out firstly in Wuhan, Hubei Province, China in December 2019, then the epidemic became prevalent in the rest of the world. With the research on COVID-19 so far, through the comparison of the gene sequence of the virus with that of the mammalian coronavirus, some studies found that its source may be related to bat, snake, mink, Malayan pangolins, turtle and other wild animals [1–4]. COVID-19 can also cause severe respiratory diseases such as fever and cough [5], and there is a possibility of transmission after symptoms of lower respiratory diseases [6]. However, unlike SARS-CoV and MERS-CoV, COVID-19 is separated from airway epithelial cells of patients [6], yet the mechanism of receptor recognition is not consistent with SARS [7]. Therefore, the pathogenicity of COVID-19 is less than that of SARS [8], and its transmissibility is higher than that of SARS [9]. In addition, this new coronavirus presents human-to-human transmission [10], and close contact could lead to group outbreaks [11]. As of July 7th, 2020, 85,359 confirmed cases and 4,648 deaths had been reported in China [12]. In addition to China, there are over 200 countries and regions in the world with a total of 11,630,898 of confirmed cases and 538,512 of deaths [12].

The outbreak of COVID-19 happened right before the Lunar New Year, which is typical Chinese Spring Festival transportation period. With a population of over 11 million, Wuhan is one of the major transportation hubs in China as well as a core city of the Yangtze River Economic Belt. The time and location of the outbreak further led to the rapid spread of the epidemic in China [13]. Since there is still no vaccine or antiviral drug specifically for COVID-19, the government's policies or actions play an important role in flatting the epidemic curve [14]. From the perspective of public health, the interventions of Wuhan government have achieved the purpose of reducing the flow of people and the risk of exposure to the diagnosed patients, and also effectively slowed down the spread of the epidemic [15]. Nevertheless, COVID-19 can be transmitted by asymptomatic carriers [16], and some of the recovered patients may still be virus carriers [17]. In order to implement non-pharmaceutical interventions more effectively, we used a combination of epidemiological methods, mathematical or statistical modeling tools to provide valuable insights and predictions as benchmarks.

For the study of infectious diseases like COVID-19, SARS, and Ebola, most of the literature used descriptive research or model methods to assess indicators and analyze the effect of interventions, such as combining migration data to evaluate the potential infection rate [18, 19], understanding the impact of factors like environmental temperature and vaccines that might be potentially linked to the diseases [20, 21], using basic and time-varying reproduction number (R_0_ & R_t_) to estimate changeable transmission dynamics of epidemic conditions [22–27], calculating and predicting the fatal risk to display any stage of outbreak [28–30], or providing suggestions and interventions from risk management and other related aspects based on the results of modeling tools or historical lessons [31–39]. Some literature only used one kind of model to simulate and predict the course of diseases. For instance, to use relatively common epidemiological dynamics models like SEIR or SIRD to forecast epidemic trends and peaks in certain provinces, even the world [9, 40–44]; to apply some other types of statistical models such as the logistic growth models or time series approaches to analyze the epidemic situation [45, 46], or to develop new models to support more complex trajectories of epidemics or to predict the number of confirmed cases and the spatial progression of outbreaks [47–49]. Several studies were further expanded based on the basic epidemic dynamic models. For example, joining the border protection mechanism with the SEIR model to better identify high-risk groups and infected cases [50]; adding the effect of media or awareness into basic models to assess whether these outside influences would possible change the transmission mode of infectious diseases [51, 52]; or according to transmission routes contained in dynamic models, using a multiplex network model or transmission network topology to analyze the outbreak scale and epidemic spread more accurately [53, 54]. A small number of studies combined the analysis capabilities of two types of models, like SEIR model and the recurrent neural networks model (RNN), to determine whether certain interventions could affect the results of outbreak control [55]. However, we did not find any analysis method using geographically weighted regression (GWR) on COVID-19 study based on our literature research. There is also a lack of understanding the model efficacy of predicting the epidemic curve among different algorithms.

In this study, an SEIR's extended model SEIRD was used to simulate the epidemic situation in China and to predict the number of confirmed and cured cases in each province and several major Chinese cities. An LSTM model combined with traffic data and a GWR model were used to predict the number of confirmed patients. Specifically, GWR Model showing geographical differences was used to predict the development of epidemic situation and analyze the impact of geographical factors. This paper also compares the characteristics and prediction ability of these models. In the absence of vaccines and drugs for COVID-19, it makes sense to use multiple models to show the situation and intensity of non-pharmaceutical interventions needed to simulate and guide the control of outbreaks.

## Materials and methods

### Data sources

Daily updated COVID-19 epidemiological data used in this study were retrieved from National Health Commission of China [12] and accessed via https://github.com/wybert/open-wuhan-ncov-illness-data. The daily number of outbound from Wuhan city and relevant migration indice from January to March were collected from an online platform called Baidu Qianxi [56]. The demographic data and medical resources data were from China urban statistical yearbook published by the National Bureau of Statistics as shown in S1 Table.

### Modified SEIRD model

**Fig 1:**
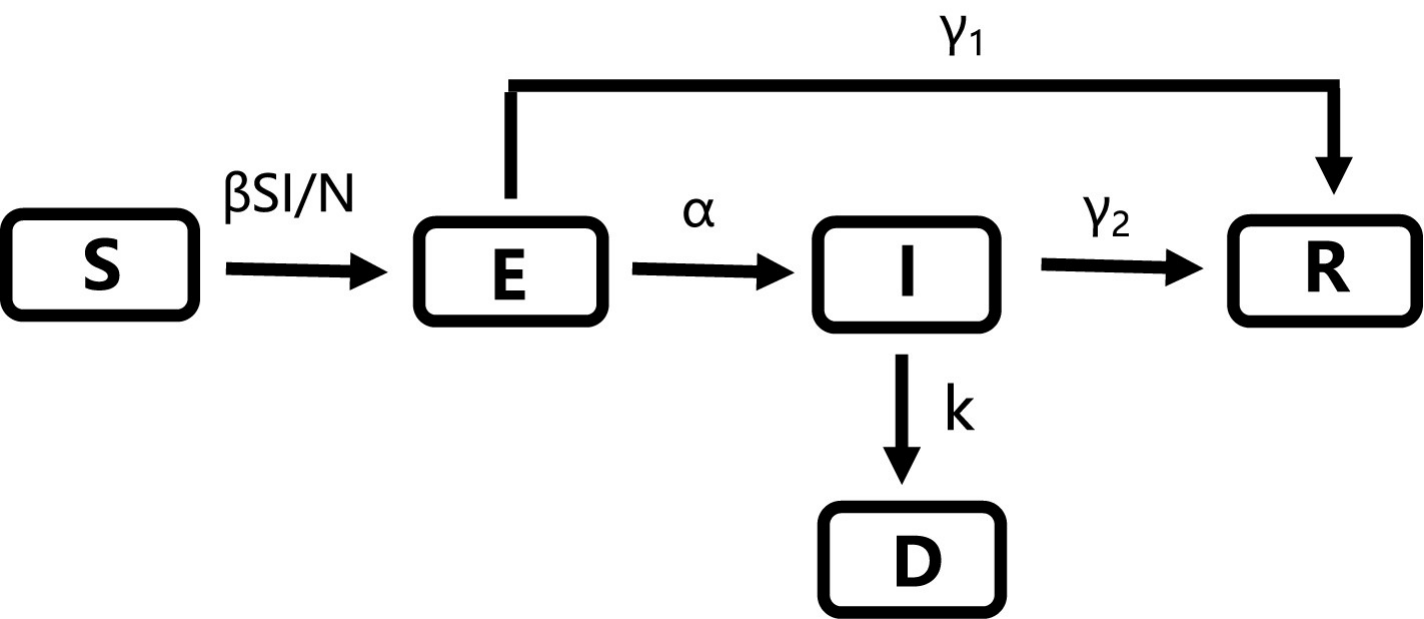
The changes of different status in the modified SEIRD model in this study.

This study used SEIRD model and the changes in the status of the susceptible (S), exposed (E), infected (I), recovered (R) and dead (D) population in the total population (N) are shown in Fig 1.

According to the medical characteristics and clinical trials of COVID-19, both confirmed patients and asymptomatic carriers have the ability to transmit the virus. Therefore, susceptible people have a certain chance to become infected after they come into contact with exposed or infected individuals [43]. Carriers in the exposed status may develop obvious symptoms after the incubation period and become diagnosed or they may be recovered. The final status of individuals can be basically divided into two categories: one is the recovery from the combined effects of treatment in hospital and autoimmunity, and the other is the death without effective treatment. In the model formula, the infectious rate *β* needs to be adjusted in real time to adapt to the trend of disease development. In the middle and late stages of the epidemic, the number of daily new cases decreased significantly due to the positive influence of government policies. Thus, to better fit the model, we added an attenuation factor desc to *β*. Based on the basic SEIRD model formulas [57, 58], our modified model was shown as Eqs (1–6).

$$\frac{dS(t)}{dt}=-\frac{\beta (t)S(t)I(t)}{N}$$ (1)

$$\frac{dE(t)}{dt}=\frac{\beta (t)S(t)I(t)}{N}-(\alpha +\gamma_{1})E(t)$$ (2)

$$\frac{dI(t)}{dt}=\alpha E(t)-\gamma_{2}I(t)-kI(t)$$ (3)

$$\frac{dR(t)}{dt}=\gamma_{1}E(t)+\gamma_{2}I(t)$$ (4)

$$\frac{dD(t)}{dt}=kI(t)$$ (5)

$$\frac{d\beta (t)}{dt}=-\beta (t)(1-desc)$$ (6)

Here, the parameter *t* denotes the time; *β* is the infectious rate; *α* is the rate for the exposed to be infected; *γ*_*1*_ is recovery rate for the exposed; *γ*_*2*_ is the recovery rate for the infected; *k* is the mortality rate; “desc” is the attenuation factor for *β*, so that *β* decays exponentially when 0<*desc*<1, and *β* is a constant when *desc* = 1.

### LSTM model

**Fig 2:**
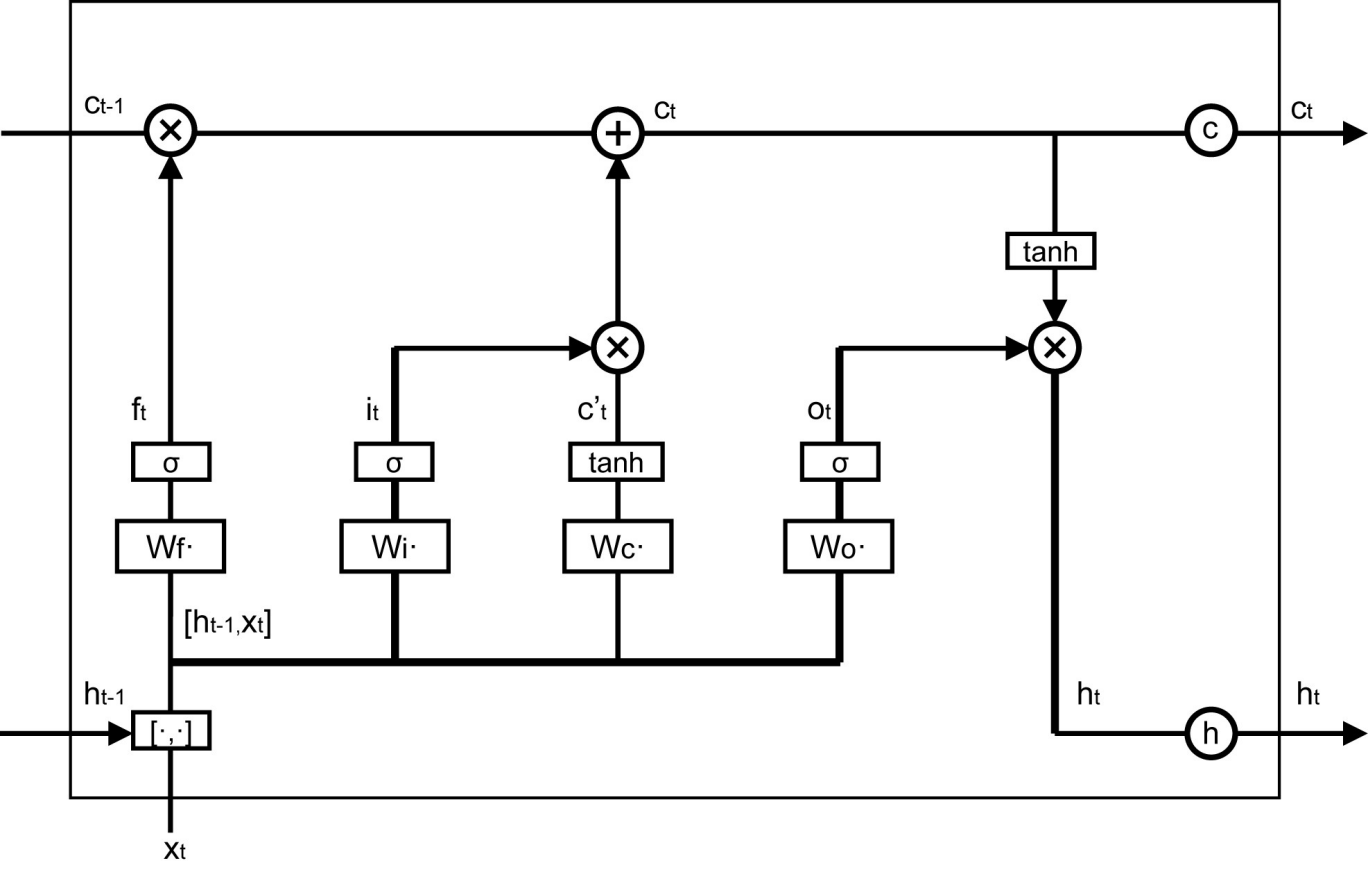
The structure of a LSTM block in this study.

LSTM (Long Short-Term Memory) architecture for recurrent neural networks was first proposed in 1997 [59]. A LSTM block is illustrated in Fig 2. It features three gates (input, forget, and output), a block input and an output. The output of the block is recurrently connected to the input of the block.

The vector formulas for a LSTM layer forward pass are given below in Eqs (7–12).

$$z_{t}=ReLU(W_{z}[x_{t},h_{t-1}]+b_{z})$$ (7)

$$i_{t}=\sigma (W_{i}[x_{t},h_{t-1}]+b_{i})$$ (8)

$$f_{t}=\sigma (W_{f}[x_{t},h_{t-1}]+b_{f})$$ (9)

$$c_{t}=i_{t}⨀z_{t}+f_{t}⨀c_{t-1}$$ (10)

$$o_{t}=\sigma (W_{o}[x_{t},h_{t-1}]+b_{o})$$ (11)

$$h_{t}=o_{t}⨀ReLU(c_{t})$$ (12)

Here, *z*_*t*_, *i*_*t*_, *f*_*t*_, *c*_*t*_, *o*_*t*_ and *h*_*t*_ denote the block input, input gate, forget gate, cell state, output gate and block output, respectively. And *x*_*t*_ represents the input vector at time *t*, ⨀ is the point-wise multiplication operator of two vectors, the *W*_*z*_, *W*_*i*_, *W*_*f*_, and *W*_*o*_ are input weight matrices, and *b*_*z*_, *b*_*i*_, *b*_*f*_, and *b*_*o*_ are bias vectors. Logistic sigmoid $\left(\sigma (x)=\frac{1}{1+e^{-x}}\right)$ is used as the activation function of the gates and *ReLU* is used as the activation function of the block input and output.

### GWR model

Epidemic situations and medical resources in different geographic situations may have different extents of influence on the development of the epidemic. Ordinary least squares fitting method for regression may not be applicable in this case. Geographically weighted regression model (GWR) was proposed in 1996 [60], which extended the ordinary linear regression model and embedded the geographic location data into the regression parameters as shown below:

$$y_{i}=\beta_{i0}+\sum_{i=0}^{p}\beta_{ik}x_{ik}+\varepsilon_{i},$$ (13)

where *y*_*i*_ is the *i*^*th*^ dependent variable, *x*_*ik*_ is the k^th^ independent variable in location *i*, *p* is the total number of independent variables, *β*_*i0*_ is the intercept parameter in location *i*, *β*_*ik*_ is the regression coefficient for the *k*^*th*^ independent variable in location *i*, which varies with the geographical location, and *ε_i_* is the error term in location *i*. The spatial weight matrix in this study uses the bi-square kernel function shown below:

$$w_{ij}={\left(1-(d_{ij}/b{)}^{2}\right)}^{2},$$ (14)

if *d*_*ij*_*<b*, otherwise *w*_*ij*_ *= 0*, where *b* is the bandwidth, a non-negative attenuation parameter and *d*_*ij*_ denotes the distance between the *i*^*th*^ and *j*^*th*^ observation points. The bandwidth is calculated by optimizing the root mean square prediction error of cross-validation [61, 62].

## Results

### SEIRD model

In this study, we used the modified SEIRD model to make predictions of the number of cumulative confirmed cases in the next day for all provinces, province-level municipalities and autonomous regions in China as well as Wuhan City. The parameters were adjusted daily in our dynamic SEIRD model based on the daily updated epidemic data. The comparison of the actual data on February 14^th^ and February 25^th^ with the forecast results of our models is shown in Table 1. The percent error was calculated using the formula: (predicted number—actual number)/ actual number × 100%. On February 14^th^, the absolute percent errors of all provinces were < 5%. The percent error for Wuhan City, Hubei Province and China were -3.00%, -1.60% and 1.00%, respectively. On February 25^th^, the absolute percent error of prediction of cumulative confirmed cases in China was < 0.10%. The absolute percent errors of most provinces were < 0.10%, among which the absolute percent errors in Wuhan City was < 0.10% and that of Hubei province was less than 0.10%. Regarding the number of recovered cases, Wuhan City and Hubei Province had percent errors of -6.03% and -3.12%, respectively. The overall prediction of recovered of the whole country was consistent with the actual situation with percent error of -2.46%. The predicted number of deaths in Hubei province was off by 1.40% (forecast 2,599 vs. actual 2,563).

**Table 1:** The comparison of predicted cumulative confirmed cases with actual data on February 14^th^ and 25^th^ in China using SEIRD model.

	February 14^th^			February 25^th^		
Regions	Predicted	Actual	Percent errors	Predicted	Actual	Percent errors
Wuhan	34902	35991	-3.00%	47014	47071	-0.12%
Anhui	952	934	1.90%	989	989	0.00%
Beijing	378	372	1.60%	400	400	0.00%
Chongqing	527	529	-0.40%	577	576	0.17%
Fujian	288	281	2.50%	293	294	-0.34%
Gansu	89	90	-1.10%	91	91	0.00%
Guangdong	1288	1261	2.10%	1348	1347	0.07%
Guangxi	237	226	4.90%	253	252	0.40%
Guizhou	142	140	1.40%	146	146	0.00%
Hainan	162	158	2.50%	168	168	0.00%
Hebei	282	283	-0.40%	311	311	0.00%
Heilongjiang	418	418	0.00%	480	480	0.00%
Henan	1213	1184	2.40%	1273	1271	0.16%
Hong Kong	53	53	0.00%	75	81	-7.41%
Hubei	51179	51986	-1.60%	64765	64786	-0.03%
Hunan	995	988	0.70%	1017	1016	0.10%
Inner Mongolia	66	65	1.50%	75	75	0.00%
Jiangsu	588	593	-0.80%	631	631	0.00%
Jiangxi	920	900	2.20%	934	934	0.00%
Jilin	88	86	2.30%	93	93	0.00%
Liaoning	123	117	5.10%	121	121	0.00%
Macau	10	10	0.00%	10	10	0.00%
Ningxia	68	67	1.50%	71	71	0.00%
Qinghai	18	18	0.00%	18	18	0.00%
Shaanxi	239	230	3.90%	245	245	0.00%
Shandong	534	523	2.10%	757	755	0.26%
Shanghai	131	126	4.00%	335	335	0.00%
Shanxi	324	318	1.90%	132	133	-0.75%
Sichuan	471	463	1.70%	528	529	-0.19%
Taiwan	18	18	0.00%	28	30	-6.67%
Tianjin	122	120	1.70%	136	135	0.74%
Tibet	1	1	0.00%	1	1	0.00%
Xinjiang	68	65	4.60%	76	76	0.00%
Yunnan	162	162	0.00%	174	174	0.00%
Zhejiang	1167	1155	1.00%	1206	1205	0.08%
China	63321	63940	-1.00%	77757	77779	-0.03%

**Fig 3:**
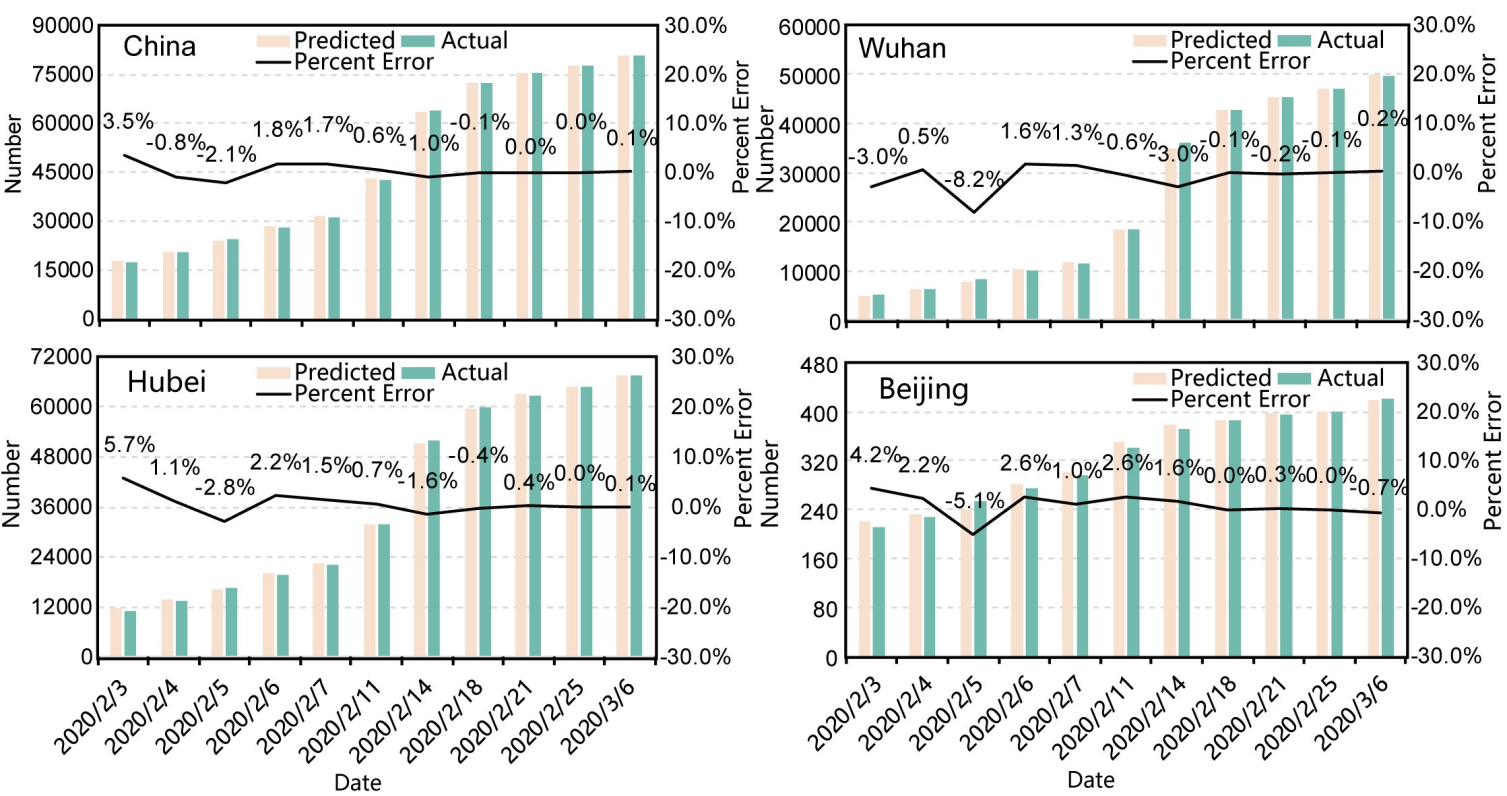
Summary of the prediction for cumulative number of COVID-19 cases and percent errors by modified SEIRD model for China, Hubei province, Wuhan city and Beijing city.

Fig 3 shows a summary of the prediction results of the cumulative number of COVID-19 cases across the country, Hubei province, Wuhan city and Beijing city by the modified SEIRD dynamics model. With the increase of the total number of cases, the percent errors in all four regions tended to decrease and the general absolute percent error in late February was ≤ 0.5%.

**Fig 4:**
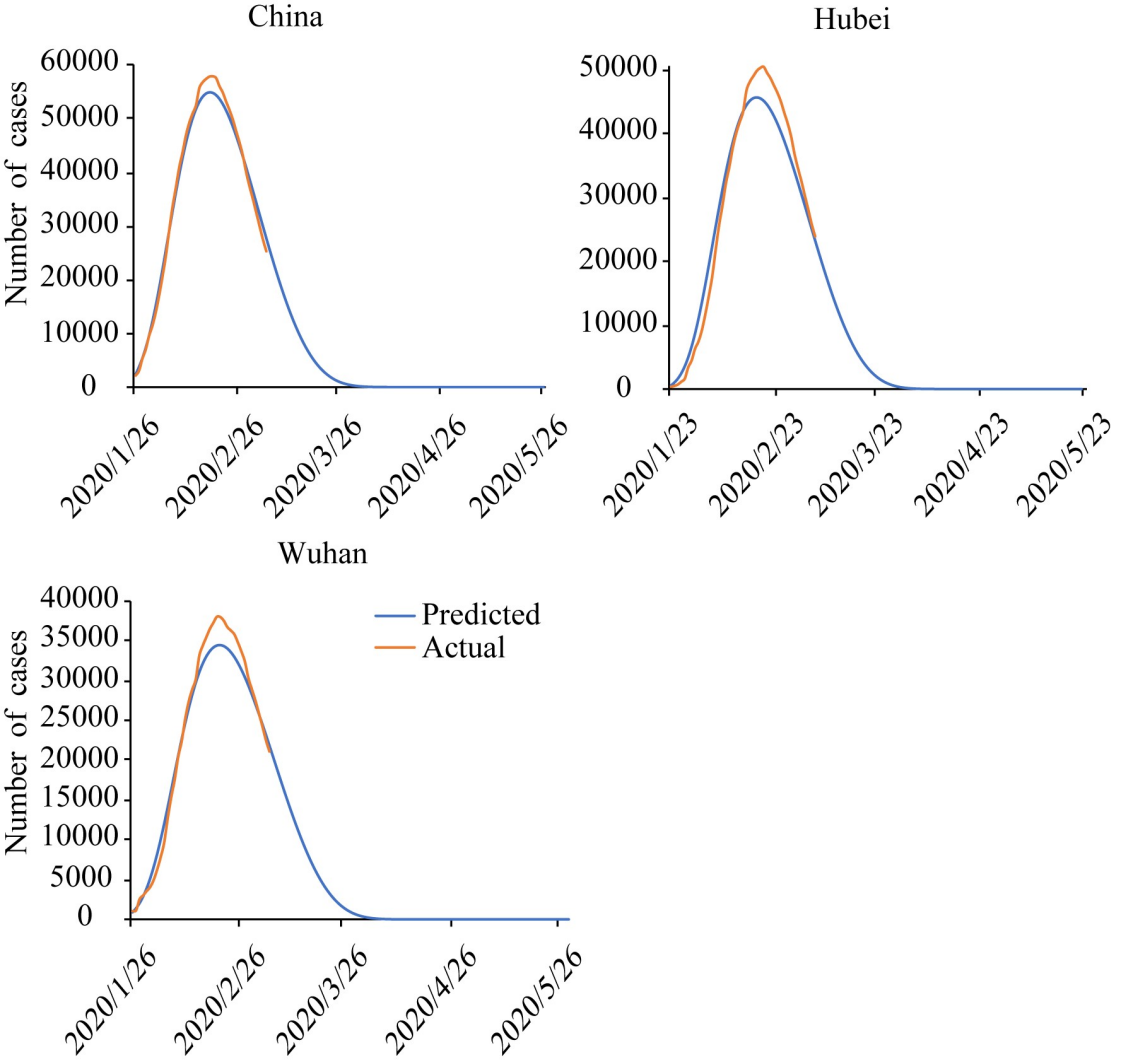
Number of actual and predicted data of existing confirmed cases by the modified SEIRD model for China, Hubei province and Wuhan city.

**Fig 5:**
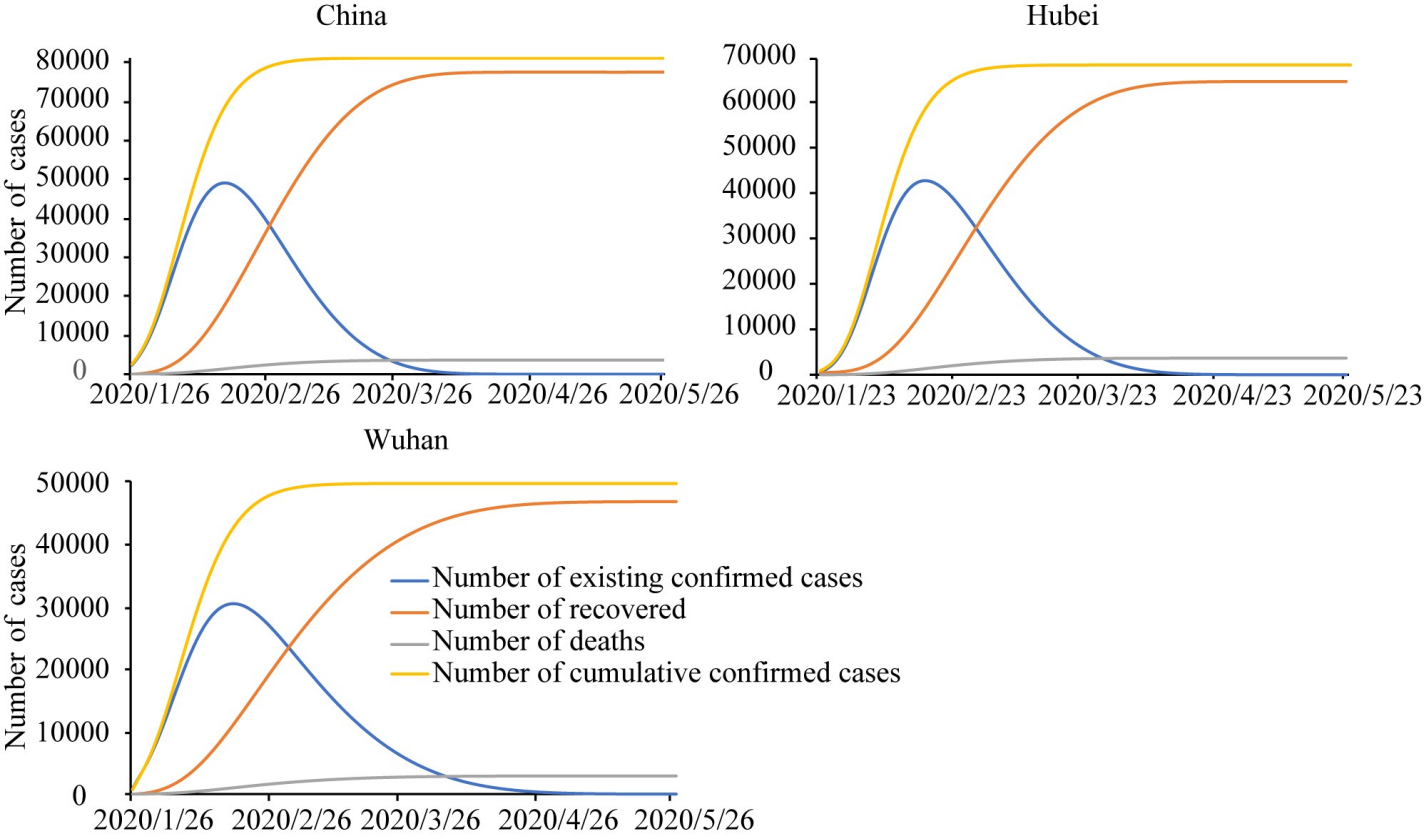
Long-term prediction of confirmed cases by the modified SEIRD model for China, Hubei province, Wuhan city and Beijing city.

Actual and predicted number of confirmed cases using the modified SEIRD model for China, Hubei province and Wuhan city are shown in Fig 4 (Hubei province and Wuhan City adjusted the criteria for diagnosis on February 13^th^, and the number of confirmed cases increased by about 10,000 on that day [63]. In order to smooth the sudden change, the number of cumulative cases before February 12^th^ in Hubei City and Wuhan province was proportionally enlarged according to the new criteria. The same for Fig 5). The actual and calculated values of these three regions provided satisfying fitting curves, indicating that the situation simulated by the model was basically in line with the actual situation of the epidemic development. In this study, the inflection point was defined as the date when the number of existing confirmed cases has the largest slope. According to the SEIRD dynamic model, the inflection points of all provinces appeared generally in February, while the specific time varied from region to region. The results of model simulation revealed that the inflection point in Wuhan city and Hubei province showed up in early February, and that of the whole country roughly in the first half of February, which basically conformed to the spread of COVID-19 in China.

Using data on March 5^th^, the model predicted the long-term trends in the number of confirmed, cured and deaths for China, Hubei province and Wuhan city (Fig 5). Again, the model used adjusted historical data as discussed above. Under the various social non-pharmaceutical interventions and not allowing for the imported cases from foreign countries, the cumulative number of confirmed nationwide was expected to reach about 83,000 at the end of the epidemic. Hubei Province was expected to have a total of about 70,000 confirmed cases and Wuhan City about 50,000.

### LSTM model

Data from four regions, Zhejiang, Guangdong, Beijing, and Shanghai were selected to train the LSTM neural network to predict the number of cumulative confirmed cases of the next day. Since the LSTM model had a memory function, the first feature included in the model was the number of cumulative confirmed cases on the previous day. Considering that the number of migrants from Wuhan also affected the studied city, thus the number of migrants from Wuhan was also included in the analysis. There was a certain probability that some migrants from Wuhan may be patients because of the virus’s incubation period, and the inference of this probability was based on the number of confirmed cases in Wuhan. Therefore, the second feature considered the number of migrants from Wuhan on the previous day, and the confirmed number of patients in Wuhan on the previous day. The feature was calculated as the cumulative number of immigrants from Wuhan multiplied by the incidence of COVID-19 in Wuhan on the previous day.

**Fig 6:**
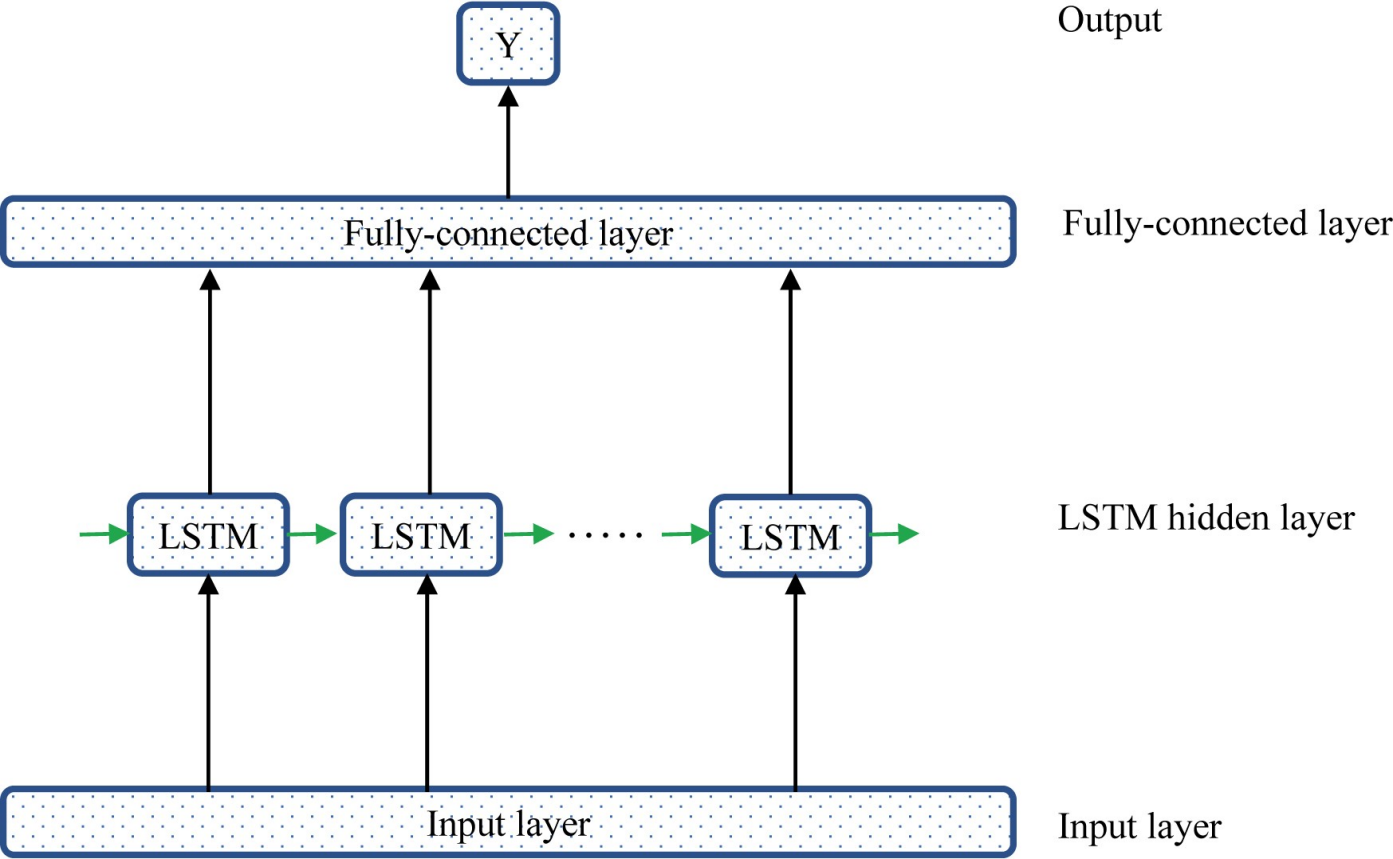
LSTM network structure of predicting COVID-19.

This LSTM architecture was designed into 4 layers: an input layer, an LSTM layer (hidden layer), a fully-connected layer and an output. Each LSTM neuron had 10 hidden features, and the activation function was *ReLU*. The loss function was MSE, and the optimizer was “Adam”. The model structure diagram is as Fig 6. This study used the grid search method to set different hyperparameters for data in different regions.

**Fig 7:**
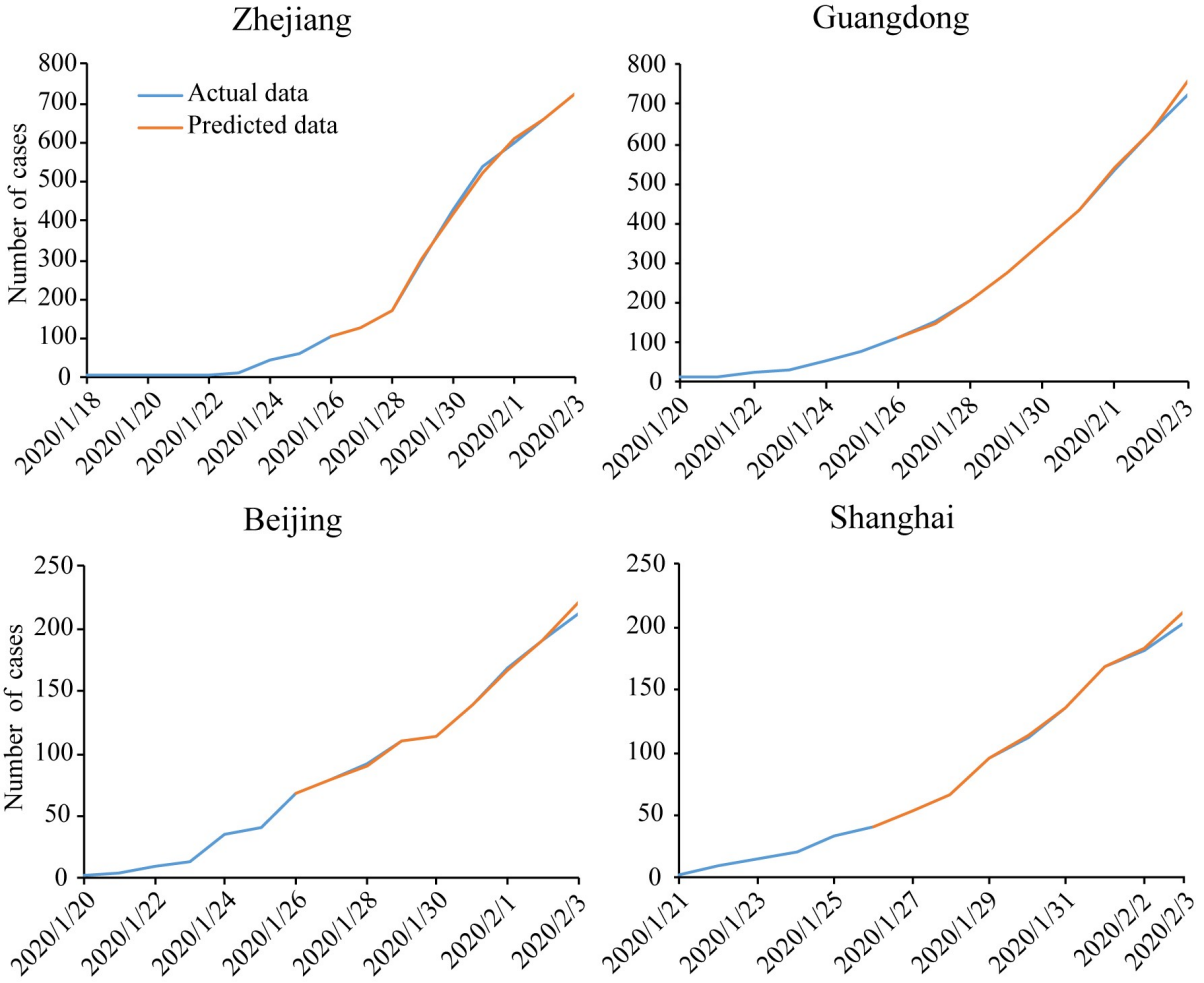
The results of prediction of cumulative confirmed cases in different regions for February 3^rd^.

**Fig 8:**
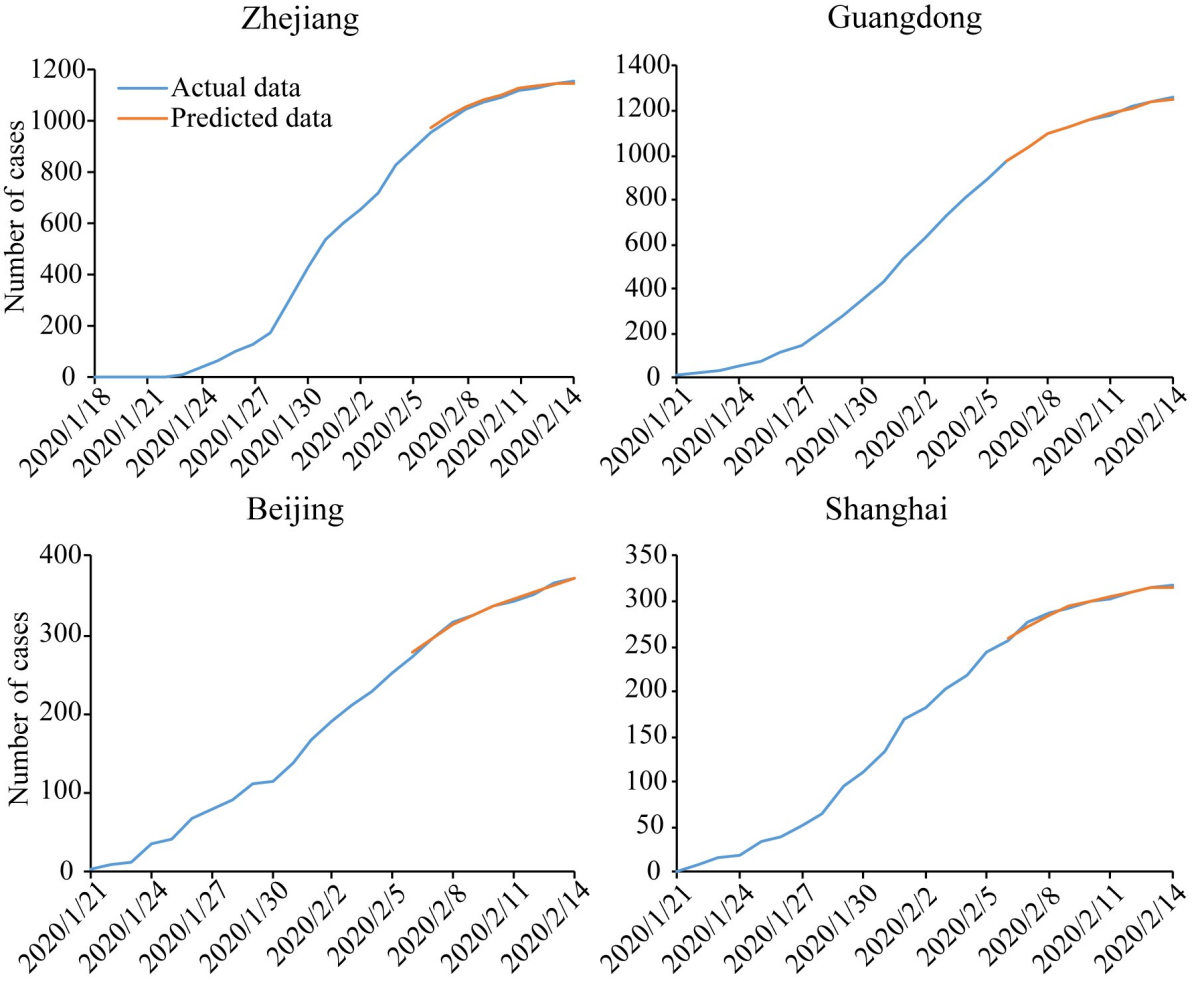
The results of prediction of cumulative confirmed cases in different regions for February 14^th^.

The model was trained and the predicted results for latest 8 consecutive days as shown in Figs 7 and 8. Finally we forecast the number of cumulative confirmed cases on the next day. The results of the forecast on February 2^nd^ (predicting the number of confirmed cases on February 3rd) and February 13^th^ (predicting the number of confirmed cases on February 14^th^) are shown in Figs 7 and 8, respectively.

The percent error is calculated as: (predicted number—actual number) / actual number ×100%. The results are shown in Tables 2 and 3. The absolute percent errors are ≤ 5.1% in all models /on February 3^rd^, and ≤ 0.63% in all models on February 14^th^.

**Table 2:** Results of the prediction of number of confirmed cases on February 3^rd^.

Area	Date	Actual number of confirmed cases	Predicted number of confirmed cases	Percent error
Zhejiang	2020/2/3	724	723	-0.14%
Guangdong	2020/2/3	725	762	5.10%
Beijing	2020/2/3	212	221	4.25%
Shanghai	2020/2/3	203	213	4.93%

**Table 3:** Results of the prediction of number of confirmed on February 14^th^.

Area	Date	Actual number of confirmed cases	Predicted number of confirmed cases	Percent error
Zhejiang	2020/2/14	1155	1151	-0.35%
Guangdong	2020/2/14	1261	1255	-0.48%
Beijing	2020/2/14	372	372	0.00%
Shanghai	2020/2/14	318	316	-0.63%

### GWR model

In this study, the data of 220 cities that had confirmed cases on February 2^nd^ were selected to predict the number of confirmed cases on February 3^rd^. The number of confirmed cases, the number of deaths and the number of cured cases are main indicators for the epidemic. Among them, the number of confirmed cases was the mostly used and reflected the severity of COVID-19 epidemic. Therefore, this study used the cumulative number of confirmed cases in different places released by the National Health Commission as dependent variable. In this study we select the population of each city, the number of hospitals per 10,000 people, the number of doctors per 10,000 people, the number of inpatient beds per 10,000 people, the number of confirmed cases, the number of cured cases, and the number of deaths one day and 2 days ago as independent variables.

The GWR model was fitted using the data of February 2^nd^, and we further made forecast for the number of the confirmed cases on February 3^rd^. The R^2^ of GWR regression on February 2^nd^ was 99.98% and the R^2^ of the prediction of the data on February 3^rd^ was 97.95%. The percent errors of fitting and prediction varied for different cities: for Beijing were 11.67% and 3.95%, respectively; for Shanghai were 2.24% and -5.88%, respectively, for Xiaogan in Hubei Province were -1.27% and 1.70%, respectively, and for Wuhan were 0.00% and 14.57%, respectively.

The summary of the intercept and coefficients of the independent variables were listed in Table 4. It shows that the coefficients of the demographic data, and the medical resources data have larger variations than those of epidemic data. The coefficients of population, number of hospitals per 10,000 people, number of doctors per 10,000 people, dead_lag1, confirmed_lag2, cured_lag2 were negative, showing that these factors have negative influence on the dependent variable. While the other independent variables, number of inpatient beds per 10,000 people, confirmed_lag1, cured_lag1, dead_lag2 have positive coefficients, indicating positive influence on the dependent variable as shown in Table 4.

**Table 4:** Summary of the coefficients of GWR model.

Variable	Min	Upper Quartile	Median	Lower Quartile	Max	Overall
Intercept	1.339	1.419	1.484	1.533	1.970	1.457
Population/10,000	-0.450	-0.419	-0.406	-0.394	-0.331	-0.400
Number of hospitals per 10,000 people	-7.512	-6.875	-6.720	-6.532	-6.124	-6.926
Number of doctors per 10,000 people	-0.193	-0.169	-0.163	-0.157	-0.145	-0.158
Number of inpatient beds per 10,000 people	0.122	0.127	0.128	0.130	0.136	0.125
Confirmed_lag1 ^a^	1.535	1.541	1.544	1.545	1.556	1.547
Cured_lag1 ^a^	6.989	7.130	7.177	7.220	7.312	7.087
Dead_lag1 ^a^	-10.902	-10.664	-10.524	-10.429	-9.787	-10.494
Confirmed _lag2 ^a^	-0.417	-0.404	-0.401	-0.398	-0.390	-0.405
Cured_lag2 ^a^	-9.417	-9.358	-9.308	-9.271	-8.994	-9.231
Dead_lag2 ^a^	14.431	15.138	15.245	15.395	15.631	15.206

^a^ Confirmed, Cured, and Dead denote the number of confirmed, cured, dead cases, respectively, and lag1 and lag2 denote one day and 2 days ago, respectively

## Discussion

### Sensitivity analysis of parameters

**Fig 9:**
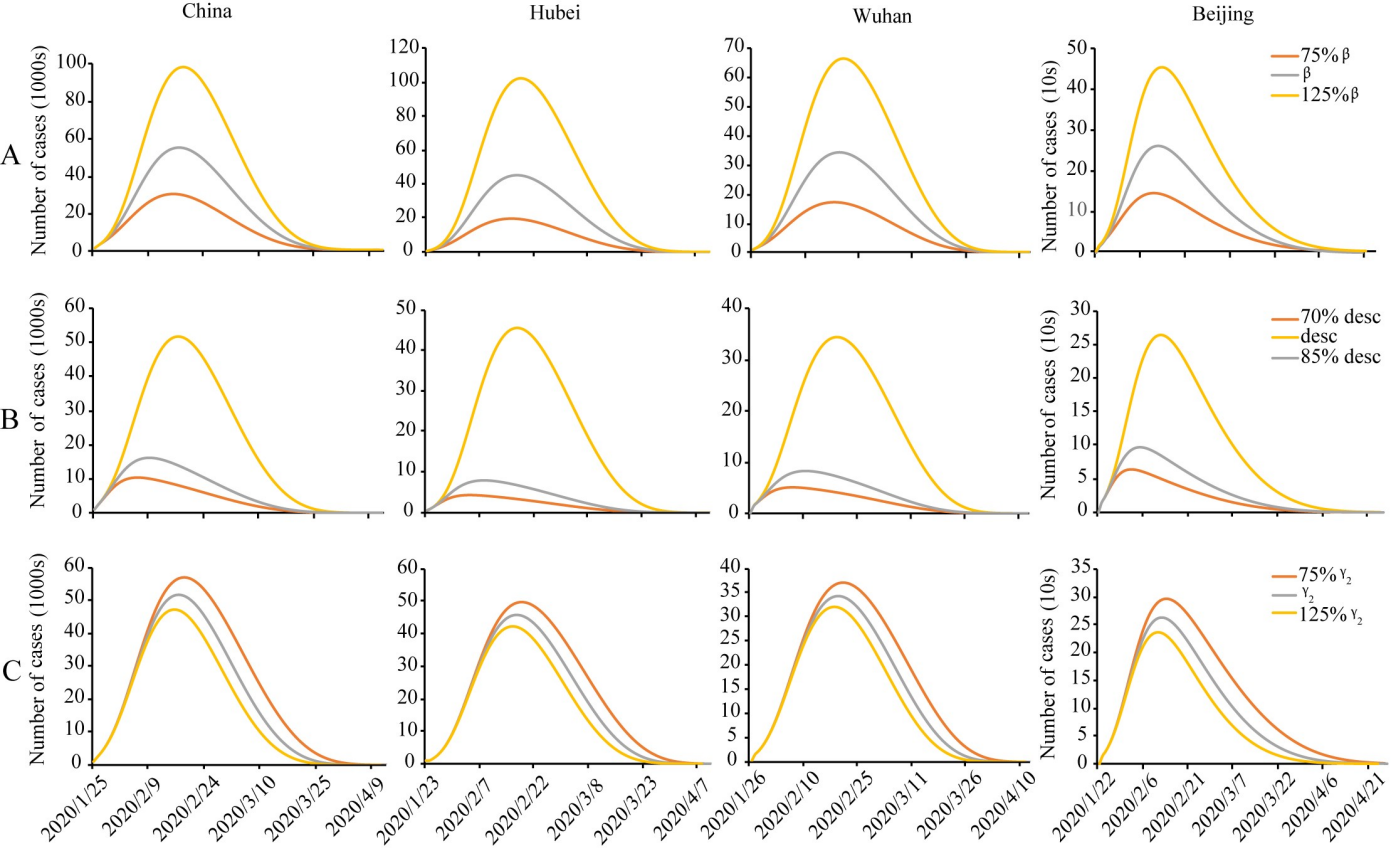
Number of infections predicted by modified SEIRD model for China, Hubei province, Wuhan city and Beijing city under different scenarios. (A) *β*, (B) *desc*, and (C) *γ*_*2*_.

As of mid-March 2020, more than 60,000 people had been cured in 31 provinces, province-level municipalities, and autonomous regions in China, and new cases of infection were mainly led by overseas imports. Although the COVID-19 epidemic was not over, the traffic in the low- and medium-risk areas in Hubei province had been gradually resuming, indicating that the government's non-pharmaceutical interventions had significantly positive effects. In this study, the modified SEIRD model was used to conduct parameter sensitivity analysis of *β*, *desc*, and *γ*_*2*_ based on data before March 5^th^, so as to simulate the impact of prevention and control measures on real-time infections for China, Hubei Province, Wuhan city, and Beijing city (Fig 9).

The decrease of the infectious rate *β* would promote the reduction of infections during the entire epidemic stage with other conditions being equal (Fig 9A). The shape of the epidemic curve was basically unchanged, but the duration of the epidemic increase as the infectious rate itself increases. The number of cases increased obviously, and the peak of real-time infections was postponed as the infectious rate increases. When the infectious rate increased to 125%, the epidemic size doubled with the delay of the peak of real-time infections by about 10 days (Fig 9A).

Moreover, increasing the attenuation factor of infectious rate could lead to a significant slowdown in the spread of the epidemic and the shape of the epidemic curve changed (Fig 9B). In the beginning, the growth of attenuation factor changed the number of confirmed cases little, but the number had changed dramatically over time, the peak of the epidemic moved forward with the increase in the attenuation factor (Fig 9B). The duration of the epidemic also advanced correspondingly. A combination of the changes in the infectious rate *β* itself and the changes in the attenuation factor of *β* could reflect the effects of the measures such as timely isolation of confirmed or suspected patients and reduction of population mobility. Coupled with the community containment measure, the number of exposed, infected and susceptible individuals outside were greatly reduced, so that the extent of the epidemic in China had been under control. Implemented metropolitan-wide quarantine of Wuhan city itself could also interfere with the change of infectious rate. The decrease in the number of daily new confirmed cases since late February showed that the corresponding policies had effectively blocked the spread of the epidemic.

The change in the recovery rate of infected *γ*_*2*_ had little effect in the early stage of the epidemic. As time went by, the growth of recovery rate could significantly raise the number of recovered, thus advancing the peak time of the real-time confirmed cases (Fig 9C). When the recovery rate raised from 75% to 125%, the whole country, Hubei province, Wuhan city and Beijing city could reach the time of maximum real-time infections about 6–15 days in advance, and the scale of the epidemic could be reduced as well (Fig 9C). In fact, China transported advantage medical resources of more than 20,000 people to Hubei province [5] in order to achieve the goal of early detection, early reporting, early diagnosis, and early isolation. Besides, the measure of “one province helping one city” established provincial counterparts to support the rescue work in Hubei province except Wuhan [5], so as to rationally allocate advanced resources. These interventions could improve the treatment and medical level of key provinces and cities, thereby increasing the recovery rate of infected and reducing the mortality rate. By March 13^th^, 2020, more than a thousand people each day have been cured and discharged for 29 consecutive days [6], indicating the effectiveness of related policies.

Although the COVID-19 has been effectively controlled in China, it has spread rapidly in other countries. Italy, the United States and Spain have become the focused areas of the outbreak. By May 2^nd^, 2020, the United States, as the country with the largest number of confirmed cases, has over 1.1 million cases, and Spain had 216,582 cases, and Italy ranked the third with 207,428 confirmed patients [12]. In order to control the spread of coronavirus, America took measures to reduce the mobility of the population, built hospitals and facilitate the treatment of the coronavirus [64–67]. Similar to the US, Italy and Spain also tried to limit the movement and gathering of the crowds, improve the protection level and provide more medical resources [64, 68–70].

In conclusion, all three countries have implemented various interventions to slow down the spread of the COVID-19 disease. The measures could be basically divided into two categories: reducing the infection rate and increasing the recovery rate. However, according to the recent large-scale outbreak in the United States and Spain, it could be found that a part of the people in these two countries might have insufficient awareness of prevention and control of the epidemic [64]. The supervision of those prevention and control measures needs further improvement. Thanks to the joint efforts of the people across the Italy, while the number of confirmed cases in Italy is still large, this country, which was called "the second Hubei province" in the early stage of the epidemic, has a trend of declining new cases of infection and death [12].

**Fig 10:**
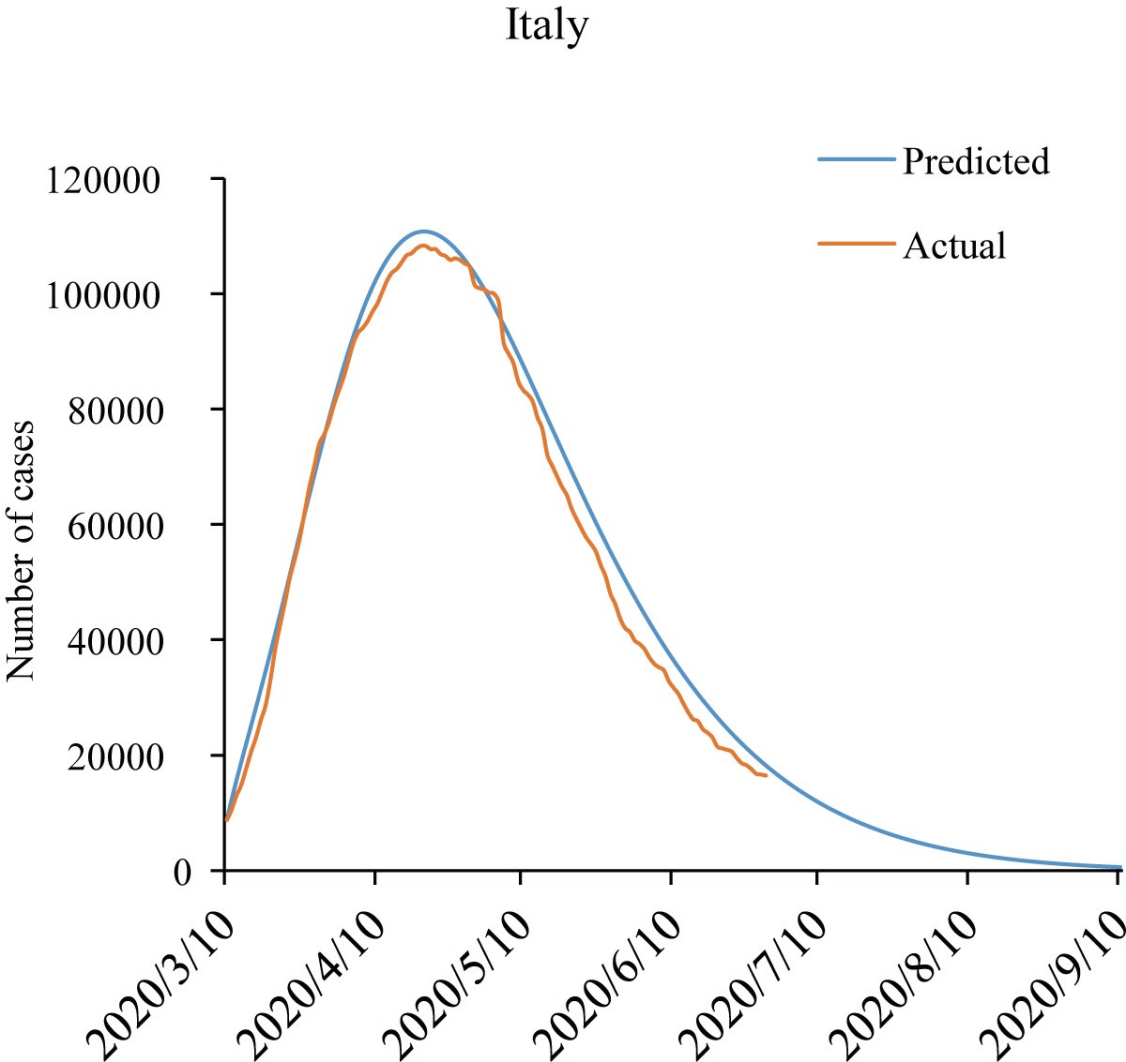
Number of actual and predicted data of existing confirmed cases by the modified SEIRD model for Italy.

In order to test the capability of the SEIRD model in foreign countries, data before June 29^th^, 2020 of Italy were used to calculate the epidemic curve. The results of the model also fitted well with the actual data as shown in Fig 10. Although some other countries successfully controlled the epidemic using similar measures with China [71], they may not always work in other countries because the effect depends on the public attitudes towards the measures and commitment to the intervention as debated in [72]. Therefore, in the face of the same epidemic situation and similar crises, our SEIRD dynamics model can be potentially applied to other countries to evaluate the intensity and effect of policies implemented by simulating and forecasting the situation of the epidemic, but the effect may be limited by the attitudes and action of the public.

### Spatial distribution of coefficients in GWR model

**Fig 11:**
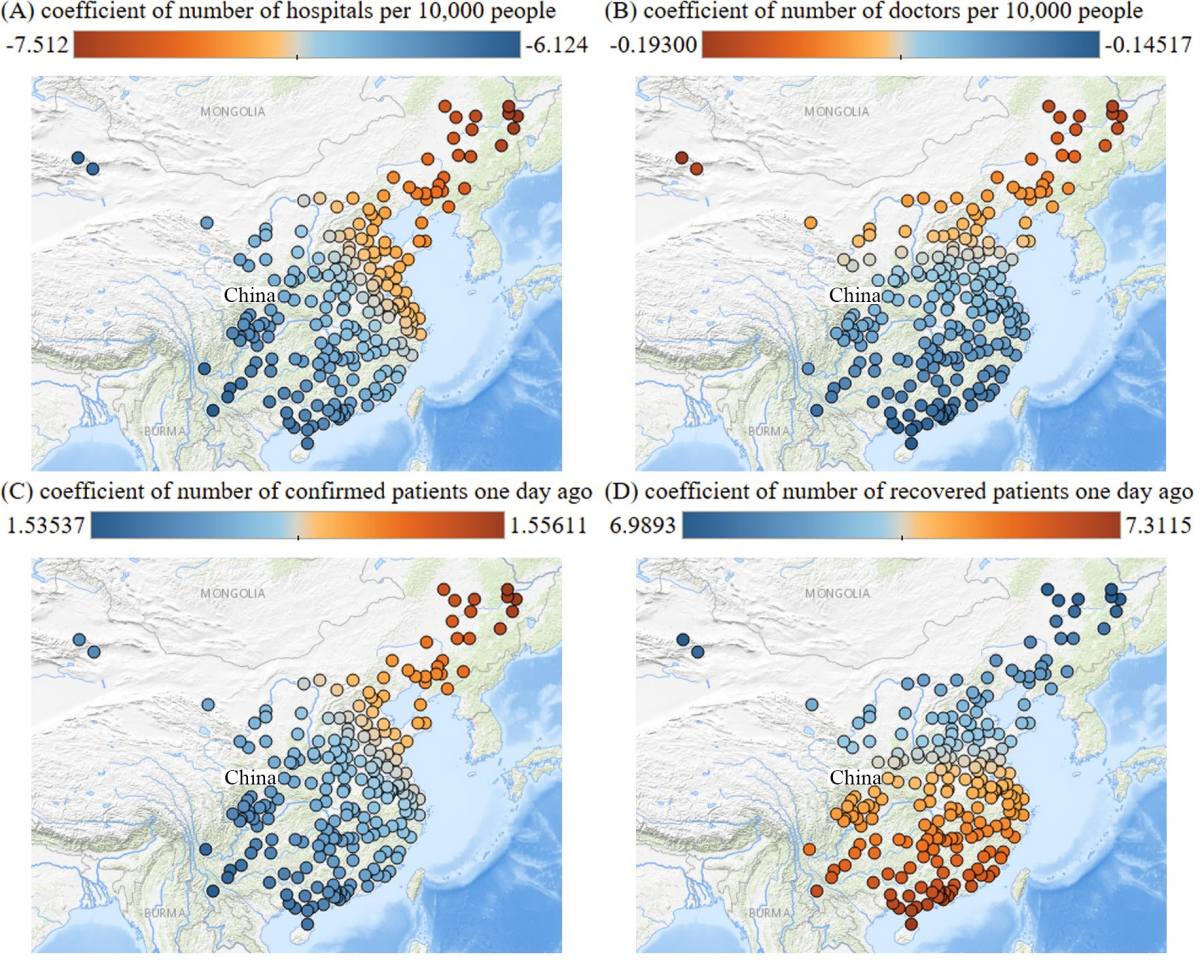
Spatial distribution of the regression coefficients in the GWR model on February 2^nd^ (the source of the maps: USGS National Map Viewer (public domain): http://viewer.nationalmap.gov/viewer/). (A) Coefficients of number of hospitals per 10,000 people. (B) Coefficients of number of doctors per 10,000 people. (C) Coefficients of number of confirmed patients one day ago. (D) Coefficients of number of recovered patients one day ago. (This figure is similar but not identical to the original image of Fig 10 in last version and is for therefore illustrative purpose only).

To better understand the spatial distribution of the coefficients of the independent variables in the GWR model, four parameters and their correlations in the model of February 2^nd^ have been studied to evaluation the heterogeneity of their coefficients in space. There was a strong negative correlation between the number of hospitals per 10,000 people and the number of confirmed cases (Fig 11A). This can be explained as that the isolation of confirmed cases in the hospital can prevent contagion. From the perspective of the spatial distribution of the regression coefficients, it has a trend of gradual decline from the northeast to the southwest and northwest of China (Fig 11A). The most influenced areas are located in the northeast of China, while the least influenced areas are in southwest and northwest of China.

There was a negative correlation between the number of doctors per 10,000 people and the number of confirmed cases (Fig 11B). From the perspective of the spatial distribution of regression coefficient, it shows a gradually decreasing trend from the northeast and northwest of China to the south (Fig 11B). The regions that are influenced the most are concentrated in northeast and northwest of China, while the least influenced regions are in the south.

There was a positive correlation between the number of confirmed cases and the confirmed cases one day ago (Fig 11C). This suggests that the more cases confirmed the day before, the more confirmed cases would emerge the next day. Effective local quarantine measures can be used to prevent a pandemic. From the perspective of the spatial distribution of the regression coefficient, it shows a trend of gradual decline from the northeast to the southwest and northwest of China (Fig 11C). This trend is not significant, which shows a universal pattern across the country.

There was a positive correlation between the number of cured case and the number of confirmed cases one day ago (Fig 11D). From the perspective of the spatial distribution of regression coefficient, it shows a gradually decreasing trend from the northeast and northwest of China to the south, with the most influenced areas in the northeast and northwest, and the least influenced areas in the south (Fig 11D).

### Comparison of SEIRD, LSTM and GWR models

By comparing the prediction capabilities of these three types of models, the modified SEIRD, LSTM and GWR model could effectively predict the epidemic data for the next day generally. The percent errors of the SEIRD model to predict confirmed cases were within ±5.0% in all of these four selected regions (Beijing, Wuhan, Hubei and China) shown in Table 5. The LSTM model also fit well to the real curve by incorporating traffic big data, indicating good simulation and prediction effects. The average percent error of LSTM model predictions for the four selected provinces and cities was within ±1.0% on February 14^th^ (Table 5). GWR model could reflect spatial heterogeneity but larger percent errors showed than the other two models in some cases (Table 5). The MAPE (Mean Absolute Percentage Error) for the SEIRD, LSTM and GWR models in the selected areas were 1.70%, 1.51%, 3.44%, respectively. In order to compare the APE (Absolute Percent Error) of the three models, we ran Wilcoxon Signed Rank Test for the paired observations in Table 5. The p-values for the hypotheses: the APE of GWR> that of LSTM, the APE of GWR > that of SEIRD and the APE of SEIRD> that of LSTM were 0.173, 0.187 and 0.459, respectively, thus not significant at the level of 0.05. Overall, the prediction efficacy of GWR model was inferior to those of SEIRD and LSTM models according to the MAPE and p-values.

**Table 5:** Comparison of the APE (Absolute percent error) of different models.

Province/City	Date	SEIRD	LSTM	GWR
Wuhan	2020/2/3	3.01%	-	14.57%
Beijing	2020/2/3	4.25%	4.25%	3.95%
Shanghai	2020/2/3	1.48%	4.93%	5.88%
Guangdong	2020/2/3	2.76%	5.10%	-
Zhejiang	2020/2/3	2.07%	0.14%	-
Wuhan	2020/2/14	3.00%	-	1.00%
Beijing	2020/2/14	3.03%	0.00%	3.62%
Shanghai	2020/2/14	1.61%	0.63%	1.17%
Guangdong	2020/2/14	1.89%	0.48%	-
Zhejiang	2020/2/14	2.14%	0.35%	-
Wuhan	2020/2/25	0.12%	-	0.14%
Beijing	2020/2/25	0.00%	0.25%	0.04%
Shanghai	2020/2/25	0.00%	0.60%	0.58%
Guangdong	2020/2/25	0.07%	0.07%	-
Zhejiang	2020/2/25	0.08%	1.33%	-

## Conclusions

In this study, the modified SEIRD model, the LSTM model with traffic data and the GWR model reflecting the geographical environment were used to make forecasts for the development of COVID–19 in China. These three types of models all showed remarkable prediction capabilities. The parameter sensitivity analysis reflected the effectiveness of non-pharmaceutical interventions. Now the epidemic quickly spread abroad, in the absence of targeted pharmaceutical treatment such as vaccines, the interventions implemented in various countries were basically similar to those in China, which were based on the two aspects: reducing the infectious rate and improving the recovery rate. As the number of daily new cases continues to increase globally, models in this study shows potential being used for epidemic curve prediction and prevention of COVID-19 in other countries.

## Supporting information

S1 Table Geographic, demographic and medical resources data for different cities. (DOCX)

## References

[1] Paraskevis D, Kostaki EG, Magiorkinis G, Panayiotakopoulos G, Sourvinos G, Tsiodras S. Full-genome evolutionary analysis of the novel corona virus (2019-nCoV) rejects the hypothesis of emergence as a result of a recent recombination event. Infect Genet Evol. 2020; 79(104212). 10.1016/j.meegid.2020.104212 32004758 PMC7106301

[2] Zhou P, Yang XL, Wang XG, Hu B, Zhang L, Zhang W, et al. Discovery of a novel coronavirus associated with the recent pneumonia outbreak in humans and its potential bat origin. bioRxiv [Preprint]. 2020 [cited 2020 Jun 27]. Available from: 10.1101/2020.01.22.914952.

[3] Wong MC, Cregeen SJJ, Ajami NJ, Petrosino JF. Evidence of recombination in coronaviruses implicating pangolin origins of nCoV-2019. bioRxiv [Preprint]. 2020 [cited 2020 Jun 27]. Available from: 10.1101/2020.02.07.939207.

[4] Zhang Z, Wu Q, Zhang T. Pangolin homology associated with 2019-nCoV. bioRxiv [Preprint]. 2020 [cited 2020 Jun 27]. Available from: 10.1101/2020.02.19.950253.

[5] Huang C, Wang Y, Li X, Ren L, Zhao J, Hu Y, et al Clinical features of patients infected with 2019 novel coronavirus in Wuhan, China. Lancet. 2020; 395(10223): 497–506. 10.1016/S0140-6736(20)30183-5 31986264 PMC7159299

[6] Perlman S. Another Decade, Another Coronavirus. N Engl J Med. 2020; 382(8): 760–762. 10.1056/NEJMe2001126 31978944 PMC7121143

[7] Tian X, Li C, Huang A, Xia S, Lu S, Shi Z, et al Potent binding of 2019 novel coronavirus spike protein by a SARS coronavirus-specific human monoclonal antibody. Emerg Microbes Infect. 2020; 9(1): 382–385. 10.1080/22221751.2020.1729069 32065055 PMC7048180

[8] Chen J. Pathogenicity and transmissibility of 2019-nCoV-A quick overview and comparison with other emerging viruses. Microbes Infect. 2020; 22(2): 69–71. 10.1016/j.micinf.2020.01.004 32032682 PMC7102641

[9] Read JM, Bridgen JR, Cummings DA, Ho A, Jewell CP. Novel coronavirus 2019-nCoV: early estimation of epidemiological parameters and epidemic predictions. medRxiv [Preprint]. 2020 [cited 2020 Jan 24]. Available from: 10.1101/2020.01.23.20018549. PMC8165596 34053269

[10] Chan JF-W, Yuan S, Kok K-H, To KK-W, Chu H, Yang J, et al A familial cluster of pneumonia associated with the 2019 novel coronavirus indicating person-to-person transmission: a study of a family cluster. Lancet. 2020; 395(10223): 514–523. 10.1016/S0140-6736(20)30154-9 31986261 PMC7159286

[11] Wu Z, McGoogan JM. Characteristics of and important lessons from the Coronavirus Disease 2019 (COVID-19) outbreak in China: summary of a report of 72 314 cases from the Chinese Center for Disease Control and Prevention. JAMA. 2020; 323(13): 1239–1242. 10.1001/jama.2020.2648 32091533

[12] Chinese National Health Commission. Reported cases of COVID-19; 2020. Available from: https://ncov.dxy.cn/ncovh5/view/pneumonia?from1⁄4groupmessage&isappinstalled1⁄40.

[13] Ai S, Zhu G, Tian F, Li H, Gao Y, Wu Y, et al. Population movement, city closure and spatial transmission of the 2019-nCoV infection in China. medRxiv [Preprint]. 2020 [cited 2020 Feb 4]. Available from: 10.1101/2020.02.04.20020339.

[14] Roosa K, Lee Y, Luo R, Kirpich A, Rothenberg R, Hyman JM, et al Real-time forecasts of the COVID-19 epidemic in China from February 5th to February 24th, 2020. Infect Dis Model. 2020; 5: 256–263. 10.1016/j.idm.2020.02.002 32110742 PMC7033348

[15] Jin G, Yu J, Han L, Duan S. The impact of traffic isolation in Wuhan on the spread of 2019-nCov. medRxiv [Preprint]. 2020 [cited 2020 Feb 24]. Available from: 10.1101/2020.02.04.20020438.

[16] Bai Y, Yao L, Wei T, Tian F, Jin D-Y, Chen L, et al Presumed Asymptomatic Carrier Transmission of COVID-19. JAMA. 2020; 323(14):1406–1407. 10.1001/jama.2020.2565 PMC7042844 32083643

[17] Lan L, Xu D, Ye G, Xia C, Wang S, Li Y, et al Positive RT-PCR Test Results in Patients Recovered From COVID-19. JAMA. 2020; 323(15):1502–1503. 10.1001/jama.2020.2783 PMC7047852 32105304

[18] Cao Z, Zhang Q, Lu X, Pfeiffer D, Wang L, Song H, et al. Incorporating Human Movement Data to Improve Epidemiological Estimates for 2019-nCoV. medRxiv [Preprint]. 2020 [cited 2020 Feb 27]. Available from: 10.1101/2020.02.07.20021071.

[19] Luo G, McHenry ML, Letterio JJ. Estimating the prevalence and risk of COVID-19 among international travelers and evacuees of Wuhan through modeling and case reports. PloS one. 2020;15(6): e0234955 10.1371/journal.pone.0234955 32574177 PMC7310725

[20] Livadiotis G. Statistical analysis of the impact of environmental temperature on the exponential growth rate of cases infected by COVID-19. PLoS ONE; 15(5): e0233875 10.1371/journal.pone.0233875 32469989 PMC7259789

[21] Lim W, Zhang P. Herd immunity and a vaccination game: An experimental study. PLoS ONE; 15(5): e0232652 10.1371/journal.pone.0232652 32407329 PMC7224512

[22] Du Z, Wang L, Cauchemez S, Xu X, Wang X, Cowling BJ, et al. Risk for Transportation of 2019 Novel Coronavirus (COVID-19) from Wuhan to Cities in China. medRxiv [Preprint]. 2020 [cited 2020 Feb 28]. Available from: 10.1101/2020.01.28.20019299.

[23] Zhang J, Litvinova M, Wang W, Wang Y, Deng X, Chen X, et al Evolving epidemiology and transmission dynamics of coronavirus disease 2019 outside Hubei province, China: a descriptive and modelling study. Lancet Infect Dis. 2020; 20(7): 793–802. 10.1016/S1473-3099(20)30230-9 32247326 PMC7269887

[24] Li MY, Smith HL, Wang L. Global dynamics of an SEIR epidemic model with vertical transmission. SIAM J Appl Math. 2001; 62(1): 58–69.

[25] Liu T, Hu JX, Xiao JP, He GH, Kang M, Rong ZH, et al. Time-varying transmission dynamics of Novel Coronavirus Pneumonia in China. bioRxiv [Preprint]. 2020 [cited 2020 Jun 27]. Available from: 10.1101/2020.01.25.919787.

[26] Huang NE, Qiao F. A data driven time-dependent transmission rate for tracking an epidemic: a case study of 2019-nCoV. Sci Bull (Beijing). 2020; 65(6): 425. 32288968 10.1016/j.scib.2020.02.005 PMC7128746

[27] Zhou Y, Ma Z, Brauer F. A discrete epidemic model for SARS transmission and control in China. Math Comput Model. 2004; 40(13): 1491–1506. 10.1016/j.mcm.2005.01.007 32288200 PMC7135158

[28] Wu P, Hao X, Lau EHY, Wong JY, Leung KSM, Wu JT, et al Real-time tentative assessment of the epidemiological characteristics of novel coronavirus infections in Wuhan, China, as at 22 January 2020. Euro Surveill. 2020; 25(3). 10.2807/1560-7917.ES.2020.25.3.2000044 31992388 PMC6988272

[29] Narayanan CS. A novel cohort analysis approach to determining the case fatality rate of COVID-19 and other infectious diseases. PLoS ONE 15(6): e0233146 10.1371/journal.pone.0233146 32542041 PMC7295185

[30] Wu JT, Leung K, Bushman M, Kishore N, Niehus R, Salazar PM, et al Estimating clinical severity of COVID-19 from the transmission dynamics in Wuhan, China. Nat Med. 2020; 26(4): 506–510. 10.1038/s41591-020-0822-7 32284616 PMC7094929

[31] Yue XG, Shao XF, Li R, Crabbe M, Mi L, Hu S, et al Risk Management Analysis for Novel Coronavirus in Wuhan, China. Journal of Risk and Financial Management. 2020; 13(2). 10.3390/jrfm13020022

[32] Riley S, Fraser C, Donnelly CA, Ghani AC, Hedley AJ, Leung GM, et al Transmission dynamics of the etiological agent of SARS in Hong Kong: impact of public health interventions. Science. 2003; 300(5627): 1961–1966 10.1126/science.1086478 12766206

[33] Kissler S M, Tedijanto C, Goldstein E, Grad YH, Lipsitch M. Projecting the transmission dynamics of SARS-CoV-2 through the postpandemic period. Science. 2020; 368(6493): 860–868. 10.1126/science.abb5793 32291278 PMC7164482

[34] Hellewell J, Abbott S, Gimma A, Bosse NI, Jarvis CI, Russell TW, et al Feasibility of controlling COVID-19 outbreaks by isolation of cases and contacts. Lancet Glob Health. 2020; 8(4): e488–e496. 10.1016/S2214-109X(20)30074-7 32119825 PMC7097845

[35] Anderson RM, Fraser C, Ghani AC, Donnelly CA, Riley S, Ferguson NM, et al Epidemiology, transmission dynamics and control of SARS: the 2002–2003 epidemic. Philos Trans R Soc Lond B Biol Sci. 2004; 359(1447): 1091–1105. 10.1098/rstb.2004.1490 15306395 PMC1693389

[36] Wallinga J, van Boven M, Lipsitch M. Optimizing infectious disease interventions during an emerging epidemic. Proc Natl Acad Sci U S A. 2010; 107(2): 923–928. 10.1073/pnas.0908491107 20080777 PMC2818907

[37] Ferretti L, Wymant C, Kendall M, Zhao L, Nurtay A, Abeler-Dörner L, et al Quantifying SARS-CoV-2 transmission suggests epidemic control with digital contact tracing. Science. 2020; 368(6491), eabb6936 10.1126/science.abb6936 32234805 PMC7164555

[38] Lipsitch M, Cohen T, Cooper B, Robins JM, Ma S, James L, et al Transmission dynamics and control of severe acute respiratory syndrome. Science. 2003; 300(5627): 1966–1970. 10.1126/science.1086616 12766207 PMC2760158

[39] Truelove S, Abrahim O, Altare C, Lauer SA, Grantz KH, Azman AS, et al The potential impact of COVID-19 in refugee camps in Bangladesh and beyond: A modeling study. PLoS Med. 17(6): e1003144 10.1371/journal.pmed.1003144 32544156 PMC7297408

[40] Wu JT, Leung K, Leung GM. Nowcasting and forecasting the potential domestic and international spread of the 2019-nCoV outbreak originating in Wuhan, China: a modelling study. Lancet. 2020; 395(10225): 689–697. 10.1016/S0140-6736(20)30260-9 32014114 PMC7159271

[41] Ai L. Modelling the epidemic trend of the 2019-nCOV outbreak in Hubei Province, China. medRxiv [Preprint]. 2020 [cited 2020 Jan 30]. Available from: 10.1101/2020.01.30.20019828.

[42] Wang H, Wang Z, Dong Y, Chang R, Xu C, Yu X, et al. Estimating the Number of 2019 Novel coronavirus cases in Chinese mainland. SSRN Electronic Journal [Preprint]. 2020 [cited 2020 Jun 27]. Available from: https://ssrn.com/abstract=3529449.

[43] Shao P, Shan Y. Beware of asymptomatic transmission: Study on 2019-nCoV prevention and control measures based on extended SEIR model. bioRxiv [Preprint]. 2020 [cited 2020 Feb 28]. Available from: 10.1101/2020.01.28.923169.

[44] Anastassopoulou C, Russo L, Tsakris A, Siettos C. Data-based analysis, modelling and forecasting of the COVID-19 outbreak. PLoS ONE. 2020; 15(3): e0230405 10.1371/journal.pone.0230405 32231374 PMC7108749

[45] Roosa K, Lee Y, Luo R, Kirpich A, Rothenberg R, Hyman JM, et al Short-term Forecasts of the COVID-19 Epidemic in Guangdong and Zhejiang, China: February 13–23, 2020. J Clin Med. 2020; 9(2). 10.3390/jcm9020596 32098289 PMC7073898

[46] Petropoulos F, Makridakis S. Forecasting the novel coronavirus COVID-19. PLoS ONE. 2020; 15(3): e0231236 10.1371/journal.pone.0231236 32231392 PMC7108716

[47] Chowell G, Tariq A, Hyman JM. A novel sub-epidemic modeling framework for short-term forecasting epidemic waves. BMC Med. 2019; 17 10.1186/s12916-019-1406-6 31438953 PMC6704534

[48] Yang W, Zhang WY, Kargbo D, Yang RF, Chen Y, Chen ZL, et al Transmission network of the 2014–2015 Ebola epidemic in Sierra Leone. J R Soc Interface. 2015; 12 10.1098/rsif.2015.0536 26559683 PMC4685836

[49] Al-Qaness MAA, Ewees AA, Fan H, Aziz MAE. Optimization method for forecasting confirmed cases of COVID-19 in China. J Clin Med. 2020; 9(3): 674. 10.3390/jcm9030674 PMC7141184 32131537

[50] Cheng ZJ, Shan J. 2019 Novel coronavirus: where we are and what we know. Infection. 2020; 48(2): 155–163. 10.1007/s15010-020-01401-y 32072569 PMC7095345

[51] Kim L, Fast SM, Markuzon N. Incorporating media data into a model of infectious disease transmission. PLoS One. 2019;14(2): e0197646 10.1371/journal.pone.0197646 30716139 PMC6361417

[52] Shang Y. Modeling epidemic spread with awareness and heterogeneous transmission rates in networks. J Biol Phys. 2013; 39(3): 489–500. 10.1007/s10867-013-9318-8 23860922 PMC3689355

[53] Zhao D, Li L, Peng H, Luo Q, Yang Y. Multiple routes transmitted epidemics on multiplex networks. Phys Lett A. 2014; 378(10): 770–776.

[54] Kamp C. Untangling the interplay between epidemic spread and transmission network dynamics. PLoS Comput Biol. 2010; 6(11): e1000984 10.1371/journal.pcbi.1000984 21124951 PMC2987842

[55] Yang Z, Zeng Z, Wang K, Wong SS, Liang W, Zanin M, et al Modified SEIR and AI prediction of the epidemics trend of COVID-19 in China under public health interventions. J Thorac Dis. 2020; 12(3): 165–174. 10.21037/jtd.2020.02.64 32274081 PMC7139011

[56] Baidu. Baidu Qianxi; 2020. Available from: https://qianxi.baidu.com/.

[57] Wu KC, Wu KL, Chen WJ, Lin MH, Li CX. Mathematical model and prediction of epidemic trend of SARS. Chin Trop Med. 2004; 3: 421–426.

[58] Pastor-Satorras R, Castellano C, Mieghem VP, Vespignani A. Epidemic processes in complex networks. Rev Mod Phys. 2015; 87: 925–979.

[59] Hochreiter S, Schmidhuber J. Long short-term memory. Neural Comput. 1997; 9: 1735–1780. 10.1162/neco.1997.9.8.1735 9377276

[60] Brunsdon C, Fotheringham AS, Charlton ME. Geographically weighted regression: a method for exploring spatial nonstationarity. Geogr Anal. 1996; 28: 281–298.

[61] Páez A, Farber S, Wheeler D. A simulation-based study of geographically weighted regression as a method for investigating spatially varying relationships. Environ Plann A. 2011; 43: 2992–3010.

[62] Fotheringham AS, Brunsdon C, Charlton ME. Geographically Weighted Regression: The Analysis of Spatially Varying Relationships. John Wiley & Sons: Chichester, England; 2002.

[63] DXY. 2019-nCoV Daily, Feb. 13; 2020. Available from: https://www.dxy.cn/bbs/newweb/pc/post/42773515?replyId=42643686.

[64] CNN. March 24 coronavirus news; 2020. Available from: https://edition.cnn.com/world/live-news/coronavirus-outbreak-03-24-20-intl-hnk/h_e735fe60223863f3f603624027a6606d.

[65] National Park Service. Active alerts in parks; 2020. Available from: https://www.nps.gov/planyourvisit/alerts.htm.

[66] Reuters. U.S. military to send field hospitals to New York, Seattle; 2020. Available from: https://www.reuters.com/article/us-health-coronavirus-usa-military/u-s-military-to-send-field-hospitals-to-new-york-seattle-idUSKBN21A357.

[67] NBCNEWS. FDA will allow doctors to treat critically ill coronavirus patients with blood from survivors; 2020. Available from: https://www.nbcnews.com/news/us-news/fda-will-allow-doctors-treat-critically-ill-coronavirus-patients-blood-n1167831.

[68] Xinhua Net. Italy under lockdown to fight coronavirus; 2020. Available from: http://www.xinhuanet.com/english/europe/2020-03/11/c_138863819.htm.

[69] Xinhua Net. Italy implements more measures in response to coronavirus epidemic; 2020. Available from: http://www.xinhuanet.com/world/2020-03/25/c_1125764995.htm.

[70] Business Insider. Spain has nationalized all of its private hospitals as the country goes into coronavirus lockdown; 2020. Available from: https://www.businessinsider.com/coronavirus-spain-nationalises-private-hospitals-emergency-covid-19-lockdown-2020-3?utm_source=yahoo.com&utm_medium=referral.

[71] Li Z, Chen Q, Feng L, Rodewald L, Xia Y, Yu H, et al Active case finding with case management: the key to tackling the COVID-19 pandemic. Lancet, 2020; 396(10243): 63–70. 10.1016/S0140-6736(20)31278-2 32505220 PMC7272157

[72] ScienceMag. China’s aggressive measures have slowed the coronavirus. They may not work in other countries; 2020. Available from: https://www.sciencemag.org/news/2020/03/china-s-aggressive-measures-have-slowed-coronavirus-they-may-not-work-other-countries.

