# Supplementary material for: Predicting and analyzing the COVID-19 epidemic in China: Based on SEIRD, LSTM and GWR models
Source: PLoS One. 2020 Aug 27;15(8):e0238280. doi: 10.1371/journal.pone.0238280 (PMC7451659; doi:10.1371/journal.pone.0238280)
Supplement: S1 Table — (DOCX) [file pone.0238280.s001.docx]

**S1 Table. Geographic, demographic and medical resources data for different cities.**

| **City** | **Longitude** | **Latitude** | **Population/10,000** | **Number of hospitals per 10,000 people** | **Number of doctors per 10,000 people** | **Number of inpatient beds per 10,000 people** |
| --- | --- | --- | --- | --- | --- | --- |
| Wuhan | 114.31 | 30.59 | 854 | 0.41 | 42.47 | 91.86 |
| Xiaogan | 113.92 | 30.92 | 519 | 0.11 | 16.92 | 28.64 |
| Huanggang | 114.87 | 30.45 | 740 | 0.09 | 18.11 | 27.97 |
| Ezhou | 114.89 | 30.39 | 111 | 0.18 | 20.14 | 40.41 |
| Jingzhou | 112.24 | 30.33 | 642 | 0.1 | 19.69 | 31.84 |
| Suizhou | 113.38 | 31.69 | 250 | 0.18 | 17.07 | 28.62 |
| Yichang | 111.29 | 30.69 | 392 | 0.23 | 27.85 | 54.36 |
| Xiangyang | 112.12 | 32.01 | 592 | 0.14 | 23.54 | 40.87 |
| Jingmen | 112.2 | 31.04 | 294 | 0.2 | 24.82 | 41.85 |
| Huangshi | 115.04 | 30.2 | 271 | 0.13 | 22.4 | 44.63 |
| Shiyan | 110.8 | 32.63 | 346 | 0.17 | 27.37 | 52.73 |
| Xianning | 114.32 | 29.84 | 304 | 0.11 | 22.18 | 31.68 |
| Shenzhen | 114.06 | 22.54 | 435 | 0.31 | 76.55 | 91.72 |
| Guangzhou | 113.26 | 23.13 | 898 | 0.27 | 55.4 | 91.03 |
| Dongguan | 113.75 | 23.02 | 211 | 0.46 | 82.97 | 137.66 |
| Foshan | 113.12 | 23.02 | 420 | 0.26 | 43.18 | 77.73 |
| Zhuhai | 113.58 | 22.27 | 119 | 0.36 | 54.01 | 70.04 |
| Huizhou | 114.42 | 23.11 | 369 | 0.2 | 34.1 | 42.14 |
| Zhongshan | 113.39 | 22.52 | 170 | 0.35 | 47.74 | 89.06 |
| Jiangmen | 113.08 | 22.58 | 396 | 0.11 | 24.9 | 41.93 |
| Zhanjiang | 110.36 | 21.27 | 839 | 0.12 | 15.27 | 30.2 |
| Zhaoqing | 112.47 | 23.05 | 446 | 0.12 | 17.09 | 28.43 |
| Maoming | 110.93 | 21.66 | 804 | 0.09 | 16.62 | 25.35 |
| Meizhou | 116.12 | 24.29 | 550 | 0.08 | 17.36 | 21.47 |
| Yangjiang | 111.98 | 21.86 | 297 | 0.17 | 17.51 | 34.07 |
| Shaoguan | 113.6 | 24.81 | 335 | 0.16 | 15.16 | 37.45 |
| Jieyang | 116.37 | 23.55 | 703 | 0.07 | 14.47 | 18.31 |
| Shanwei | 115.38 | 22.79 | 363 | 0.09 | 14.2 | 17.46 |
| Heyuan | 114.7 | 23.74 | 373 | 0.14 | 15.39 | 21.24 |
| Qingyuan | 113.06 | 23.68 | 437 | 0.13 | 17.15 | 25.18 |
| Chaozhou | 116.62 | 23.66 | 276 | 0.11 | 16.67 | 17.86 |
| Wenzhou | 120.7 | 27.99 | 825 | 0.17 | 32.33 | 44.03 |
| Taizhou | 121.42 | 28.66 | 604 | 0.18 | 28.53 | 41.32 |
| Ningbo | 121.55 | 29.87 | 597 | 0.26 | 40.65 | 57.18 |
| Hangzhou | 120.16 | 30.27 | 754 | 0.4 | 55.48 | 93.09 |
| Jiaxing | 120.76 | 30.75 | 356 | 0.21 | 32 | 61.92 |
| Jinhua | 119.65 | 29.08 | 486 | 0.27 | 33.69 | 55.17 |
| Shaoxing | 120.58 | 30.03 | 446 | 0.17 | 34.07 | 47.63 |
| Zhoushan | 122.21 | 29.99 | 97 | 0.31 | 36.92 | 52.12 |
| Quzhou | 118.87 | 28.94 | 258 | 0.29 | 26.86 | 45.04 |
| Lishui | 119.92 | 28.47 | 269 | 0.2 | 29.42 | 44.77 |
| Huzhou | 120.09 | 30.89 | 266 | 0.22 | 30.38 | 52.49 |
| Jining | 116.59 | 35.41 | 883 | 0.19 | 24.47 | 41.26 |
| Yantai | 121.45 | 37.46 | 654 | 0.3 | 27.67 | 48.63 |
| Jinan | 117.12 | 36.65 | 644 | 0.37 | 45.09 | 73.87 |
| Weifang | 119.16 | 36.71 | 908 | 0.21 | 28.01 | 44.81 |
| Qingdao | 120.38 | 36.07 | 803 | 0.51 | 38.44 | 66.43 |
| Dezhou | 116.36 | 37.44 | 595 | 0.17 | 21.87 | 30.14 |
| Weihai | 122.12 | 37.51 | 256 | 0.2 | 31.63 | 54.95 |
| Liaocheng | 115.99 | 36.46 | 640 | 0.23 | 20 | 37.74 |
| Zibo | 118.05 | 36.81 | 433 | 0.36 | 33.38 | 55.48 |
| Taian | 117.09 | 36.2 | 571 | 0.18 | 23.7 | 43.7 |
| Linyi | 118.36 | 35.1 | 1162 | 0.16 | 18.32 | 34.07 |
| Zaozhuang | 117.32 | 34.81 | 418 | 0.19 | 22.81 | 44.61 |
| Rizhao | 119.53 | 35.42 | 304 | 0.17 | 20.39 | 31.7 |
| Binzhou | 117.97 | 37.38 | 394 | 0.28 | 24.61 | 39.89 |
| Heze | 115.48 | 35.23 | 1019 | 0.23 | 20.96 | 34.02 |
| Hefei | 117.23 | 31.82 | 743 | 0.23 | 28.22 | 57.48 |
| Fuyang | 115.81 | 32.89 | 1070 | 0.11 | 13 | 26.57 |
| Bengbu | 117.39 | 32.92 | 381 | 0.21 | 16.53 | 40.13 |
| Bozhou | 115.78 | 33.84 | 651 | 0.1 | 9.83 | 19.27 |
| Lu'an | 116.52 | 31.73 | 588 | 0.05 | 15.41 | 20.92 |
| Ma'anshan | 118.51 | 31.67 | 229 | 0.27 | 20.77 | 33.23 |
| Anqing | 117.06 | 30.54 | 531 | 0.13 | 16.27 | 29.25 |
| Suzhou | 116.96 | 33.65 | 656 | 0.12 | 14.06 | 23.5 |
| Huaibei | 116.8 | 33.95 | 217 | 0.32 | 20.85 | 42.59 |
| Huainan | 117 | 32.63 | 390 | 0.18 | 15.9 | 34.99 |
| Wuhu | 118.43 | 31.35 | 388 | 0.21 | 22.43 | 45.72 |
| Chizhou | 117.49 | 30.66 | 162 | 0.19 | 18.1 | 32.02 |
| Chuzhou | 118.32 | 32.3 | 454 | 0.14 | 14.59 | 30.22 |
| Huangshan | 118.34 | 29.72 | 148 | 0.22 | 22.96 | 43.03 |
| Xuancheng | 118.76 | 30.94 | 280 | 0.16 | 19.09 | 35.87 |
| Xinyang | 114.09 | 32.15 | 911 | 0.3 | 10.91 | 26.88 |
| Zhengzhou | 113.62 | 34.75 | 842 | 0.26 | 45.19 | 94.69 |
| Nanyang | 112.53 | 32.99 | 1200 | 0.09 | 14.99 | 28.03 |
| Zhoukou | 114.7 | 33.63 | 1258 | 0.11 | 13.05 | 21.99 |
| Zhumadian | 114.02 | 33.01 | 961 | 0.08 | 13.42 | 25.37 |
| Shangqiu | 115.66 | 34.41 | 987 | 0.08 | 14.95 | 24.66 |
| Pingdingshan | 113.19 | 33.77 | 567 | 0.15 | 20.03 | 38.65 |
| Xuchang | 113.85 | 34.04 | 508 | 0.2 | 19.46 | 35.45 |
| Anyang | 114.39 | 36.1 | 624 | 0.16 | 21.25 | 34.53 |
| Luohe | 114.02 | 33.58 | 267 | 0.18 | 19.58 | 39.92 |
| Luoyang | 112.45 | 34.62 | 737 | 0.19 | 23.86 | 48.49 |
| Kaifeng | 114.31 | 34.8 | 559 | 0.19 | 20.18 | 40.2 |
| Hebi | 114.3 | 35.75 | 170 | 0.25 | 22.62 | 46.43 |
| Jiaozuo | 113.24 | 35.22 | 371 | 0.25 | 24.24 | 46.44 |
| Puyang | 115.03 | 35.76 | 432 | 0.14 | 18.38 | 32.97 |
| Xinxiang | 113.93 | 35.3 | 647 | 0.18 | 22.1 | 41.43 |
| Sanmenxia | 111.2 | 34.77 | 228 | 0.22 | 23.23 | 47.99 |
| Changsha | 112.94 | 28.23 | 709 | 0.4 | 41.28 | 83.07 |
| Yueyang | 113.13 | 29.36 | 567 | 0.43 | 26.99 | 38.59 |
| Zhuzhou | 113.13 | 27.83 | 403 | 0.47 | 26.35 | 64.51 |
| Changde | 111.7 | 29.03 | 606 | 0.14 | 25.36 | 35.53 |
| Loudi | 111.99 | 27.7 | 454 | 0.19 | 21.03 | 39 |
| Shaoyang | 111.47 | 27.24 | 826 | 0.35 | 15.5 | 32.25 |
| Hengyang | 112.57 | 26.89 | 800 | 0.16 | 22.2 | 35.97 |
| Xiangtan | 112.94 | 27.83 | 288 | 0.22 | 26.13 | 51.77 |
| Chenzhou | 113.01 | 25.77 | 534 | 0.63 | 20.65 | 42.46 |
| Yiyang | 112.36 | 28.55 | 479 | 0.17 | 22.96 | 36.84 |
| Yongzhou | 111.61 | 26.42 | 642 | 0.2 | 19.43 | 40.5 |
| Huaihua | 110 | 27.57 | 522 | 0.76 | 22.24 | 45.13 |
| Zhangjiajie | 110.48 | 29.12 | 170 | 0.18 | 18.86 | 37.02 |
| Jiujiang | 116 | 29.71 | 520 | 0.12 | 19.28 | 31.47 |
| Nanchang | 115.86 | 28.68 | 525 | 0.22 | 26.94 | 52 |
| Shangrao | 117.94 | 28.45 | 783 | 0.19 | 14.18 | 31 |
| Yichun | 114.42 | 27.81 | 602 | 0.07 | 13.46 | 22.98 |
| Ganzhou | 114.93 | 25.83 | 974 | 0.09 | 14.42 | 43.19 |
| Xinyu | 114.92 | 27.82 | 122 | 0.11 | 34.43 | 36.08 |
| Fuzhou | 116.36 | 27.95 | 431 | 0.11 | 12.83 | 22.81 |
| Pingxiang | 113.85 | 27.62 | 200 | 0.16 | 22.17 | 40.13 |
| Ji'an | 114.99 | 27.11 | 536 | 0.11 | 14.98 | 28.07 |
| Yingtan | 117.07 | 28.26 | 128 | 0.27 | 20.2 | 41.48 |
| Jingdezhen | 117.18 | 29.27 | 169 | 0.18 | 18.62 | 42.63 |
| Chengdu | 104.06 | 30.57 | 1435 | 0.62 | 40.53 | 88.81 |
| Dazhou | 107.47 | 31.21 | 672 | 0.59 | 10.32 | 42.93 |
| Nanchong | 106.11 | 30.84 | 733 | 0.15 | 17.84 | 40.5 |
| Luzhou | 105.44 | 28.87 | 510 | 0.27 | 18.11 | 39.32 |
| Bazhong | 106.75 | 31.87 | 376 | 0.2 | 17.57 | 31.97 |
| Deyang | 104.4 | 31.13 | 388 | 0.22 | 23.07 | 40.13 |
| Guang'an | 106.63 | 30.46 | 465 | 0.14 | 8.5 | 27.02 |
| Mianyang | 104.68 | 31.47 | 537 | 0.17 | 21.86 | 44.03 |
| Neijiang | 105.06 | 29.58 | 415 | 0.17 | 13.27 | 38.75 |
| Suining | 105.59 | 30.53 | 370 | 0.19 | 17.69 | 36.91 |
| Yibin | 104.64 | 28.75 | 555 | 0.31 | 16.26 | 43.45 |
| Meishan | 103.85 | 30.08 | 345 | 0.26 | 17.86 | 36.7 |
| Ya'an | 103.04 | 30.01 | 154 | 0.29 | 25.59 | 67.92 |
| Ziyang | 104.63 | 30.13 | 349 | 0.12 | 14.74 | 31.47 |
| Zigong | 104.78 | 29.34 | 324 | 0.21 | 13.98 | 46.9 |
| Panzhihua | 101.72 | 26.58 | 109 | 0.22 | 36.39 | 82.61 |
| Guangyuan | 105.84 | 32.44 | 303 | 0.24 | 19.43 | 50.53 |
| Leshan | 103.77 | 29.55 | 352 | 0.28 | 21.41 | 45.77 |
| Harbin | 126.54 | 45.8 | 955 | 0.32 | 23.04 | 73.82 |
| Shuangyashan | 131.16 | 46.65 | 94 | 0.57 | 20.98 | 89.49 |
| Jixi | 130.97 | 45.3 | 175 | 0.39 | 24.96 | 63.63 |
| Qiqihar | 123.92 | 47.35 | 534 | 0.22 | 19.43 | 47.23 |
| Daqing | 125.11 | 46.6 | 273 | 0.41 | 36.36 | 59.62 |
| Suihua | 126.97 | 46.65 | 528 | 0.12 | 20.1 | 0 |
| Qitaihe | 131 | 45.77 | 79 | 0.34 | 22.65 | 50.11 |
| Mudanjiang | 129.63 | 44.55 | 255 | 0.31 | 30.58 | 61.57 |
| Heihe | 127.53 | 50.25 | 161 | 0.39 | 25.03 | 45.28 |
| Hegang | 130.3 | 47.35 | 101 | 0.49 | 30.37 | 83.6 |
| Yichun | 128.84 | 47.73 | 116 | 0.34 | 23.14 | 54.47 |
| Jiamusi | 130.32 | 46.8 | 235 | 0.39 | 24.23 | 61.07 |
| Chongqing | 106.55 | 29.56 | 3390 | 0.22 | 20.22 | 44.4 |
| Beijing | 116.41 | 39.9 | 1359 | 0.48 | 69.48 | 83.66 |
| Nanjing | 118.8 | 32.06 | 681 | 0.32 | 41.26 | 68.96 |
| Suzhou | 120.58 | 31.3 | 691 | 0.28 | 43.85 | 81.24 |
| Lianyungang | 119.22 | 34.6 | 533 | 0.15 | 21.58 | 31.46 |
| Huaian | 119.02 | 33.61 | 561 | 0.11 | 22.64 | 31.86 |
| Wuxi | 120.31 | 31.49 | 493 | 0.34 | 39.78 | 74.13 |
| Xuzhou | 117.29 | 34.2 | 1039 | 0.13 | 22.04 | 38.27 |
| Changzhou | 119.97 | 31.81 | 379 | 0.18 | 34.56 | 55.54 |
| Taizhou | 119.93 | 32.46 | 505 | 0.14 | 22.97 | 35.5 |
| Nantong | 120.89 | 31.98 | 764 | 0.29 | 24.61 | 43.56 |
| Yangzhou | 119.41 | 32.39 | 460 | 0.15 | 23.7 | 35.35 |
| Yancheng | 120.16 | 33.35 | 826 | 0.2 | 22.03 | 36.04 |
| Suqian | 118.28 | 33.96 | 591 | 0.39 | 19.46 | 45.85 |
| Zhenjiang | 119.43 | 32.19 | 271 | 0.18 | 29.89 | 40.07 |
| Beihai | 109.12 | 21.48 | 175 | 0.14 | 20.08 | 33.1 |
| Hechi | 108.09 | 24.69 | 430 | 0.08 | 14.62 | 27.16 |
| Nanning | 108.37 | 22.82 | 757 | 0.15 | 30.93 | 47.06 |
| Liuzhou | 109.42 | 24.33 | 387 | 0.17 | 26.69 | 46.23 |
| Fangchenggang | 108.35 | 21.69 | 98 | 0.14 | 20.84 | 26.89 |
| Laibin | 109.22 | 23.75 | 268 | 0.08 | 14.07 | 22.56 |
| Guilin | 110.29 | 25.27 | 534 | 0.12 | 21.75 | 29.47 |
| Yulin | 110.18 | 22.65 | 724 | 0.06 | 12.59 | 21.2 |
| Guigang | 109.6 | 23.11 | 556 | 0.09 | 11.85 | 18.72 |
| Qinzhou | 108.65 | 21.98 | 411 | 0.05 | 12.93 | 21 |
| Baise | 106.62 | 23.9 | 418 | 0.08 | 15.27 | 26.38 |
| Wuzhou | 111.28 | 23.48 | 349 | 0.11 | 17.03 | 28.62 |
| Hezhou | 111.57 | 24.4 | 244 | 0.11 | 14.14 | 21.3 |
| Quanzhou | 118.68 | 24.87 | 742 | 0.18 | 21.43 | 34.55 |
| Fuzhou | 119.3 | 26.07 | 693 | 0.17 | 29.46 | 43.51 |
| Xiamen | 118.09 | 24.48 | 231 | 0.22 | 55 | 61.06 |
| Nanping | 118.12 | 27.33 | 319 | 0.16 | 16.87 | 38.28 |
| Ningde | 119.55 | 26.67 | 351 | 0.13 | 15.54 | 29.44 |
| Zhangzhou | 117.65 | 24.51 | 514 | 0.15 | 20.15 | 33.45 |
| Sanming | 117.64 | 26.26 | 288 | 0.16 | 18.89 | 35.99 |
| Longyan | 117.02 | 25.08 | 316 | 0.15 | 18.97 | 52.58 |
| Xi'an | 108.94 | 34.34 | 906 | 0.36 | 34.02 | 64.26 |
| Hanzhong | 107.02 | 33.07 | 382 | 0.21 | 18.3 | 45.78 |
| Ankang | 109.03 | 32.68 | 305 | 0.16 | 28.12 | 33.96 |
| Weinan | 109.51 | 34.5 | 556 | 0.28 | 17.28 | 36.75 |
| Xianyang | 108.71 | 34.33 | 468 | 0.32 | 23.12 | 49.61 |
| Tongchuan | 108.95 | 34.9 | 83 | 0.57 | 30.18 | 66.64 |
| Shangluo | 109.94 | 33.87 | 253 | 0.71 | 17.24 | 46.5 |
| Baoji | 107.24 | 34.36 | 381 | 0.28 | 25.87 | 52.57 |
| Yan'an | 109.49 | 36.59 | 238 | 0.26 | 22.13 | 42.66 |
| Yulin | 109.73 | 38.29 | 385 | 0.28 | 19.19 | 42.49 |
| Shanghai | 121.47 | 31.23 | 1455 | 0.25 | 46.67 | 79.67 |
| Tangshan | 118.18 | 39.63 | 755 | 0.23 | 24.75 | 44.32 |
| Cangzhou | 116.84 | 38.3 | 778 | 0.2 | 24.72 | 40.45 |
| Shijiazhuang | 114.51 | 38.04 | 973 | 0.24 | 35.44 | 47.31 |
| Handan | 114.54 | 36.63 | 1051 | 0.2 | 19.82 | 33.36 |
| Xingtai | 114.5 | 37.07 | 790 | 0.22 | 22.19 | 33.74 |
| Zhangjiakou | 114.89 | 40.82 | 465 | 0.22 | 20.22 | 39.9 |
| Langfang | 116.68 | 39.54 | 474 | 0.32 | 25.06 | 35.68 |
| Qinhuangdao | 119.6 | 39.94 | 298 | 0.24 | 31.7 | 46.23 |
| Baoding | 115.46 | 38.87 | 1199 | 0.3 | 23.6 | 37.02 |
| Hengshui | 115.67 | 37.74 | 454 | 0.28 | 24.25 | 35.11 |
| Chengde | 117.96 | 40.95 | 380 | 0.21 | 25.61 | 46.32 |
| Kunming | 102.83 | 24.88 | 563 | 0.55 | 48.52 | 94.27 |
| Zhaotong | 103.72 | 27.34 | 619 | 0.48 | 12.4 | 39.59 |
| Qujing | 103.8 | 25.49 | 661 | 0.17 | 14.14 | 36.48 |
| Baoshan | 99.16 | 25.11 | 263 | 0.19 | 16.58 | 33.89 |
| Yuxi | 102.55 | 24.35 | 219 | 0.33 | 26.89 | 50.77 |
| Lijiang | 100.23 | 26.86 | 123 | 0.24 | 16.95 | 36.2 |
| Pu'er | 100.97 | 22.83 | 253 | 0.17 | 16.66 | 33.99 |
| Lincang | 100.09 | 23.88 | 239 | 0.22 | 13.87 | 34.28 |
| Tianjin | 117.2 | 39.09 | 1050 | 0.41 | 39.17 | 57.29 |
| Urumqi | 87.62 | 43.83 | 223 | 0.57 | 64.94 | 123.16 |
| Turpan | 89.19 | 42.95 | 64 | 0.34 | 24.84 | 45.59 |
| Zunyi | 106.93 | 27.73 | 805 | 0.24 | 17.93 | 45.95 |
| Guiyang | 106.63 | 26.65 | 408 | 0.45 | 41.32 | 76.88 |
| Bijie | 105.31 | 27.3 | 923 | 0.55 | 12.46 | 38.07 |
| Liupanshui | 104.83 | 26.59 | 342 | 0.32 | 14.93 | 36.73 |
| Anshun | 105.95 | 26.25 | 301 | 0.24 | 12.44 | 32.31 |
| Tongren | 109.18 | 27.69 | 440 | 0.23 | 14.62 | 27.77 |
| Sanya | 109.51 | 18.25 | 59 | 0.19 | 37.61 | 40.02 |
| Haikou | 110.2 | 20.04 | 171 | 0.72 | 72.61 | 75.2 |
| Danzhou | 109.58 | 19.52 | 96 | 0.23 | 17.5 | 33.38 |
| Baotou | 109.84 | 40.66 | 224 | 0.38 | 38.34 | 70.62 |
| Chifeng | 118.89 | 42.26 | 460 | 0.21 | 25.22 | 44.31 |
| Tongliao | 122.24 | 43.65 | 316 | 0.26 | 22.51 | 45.41 |
| Hohhot | 111.75 | 40.84 | 243 | 0.44 | 42.08 | 73.2 |
| Hulunbeier | 119.77 | 49.21 | 260 | 0.84 | 37.01 | 53.28 |
| Ordos | 109.78 | 39.61 | 161 | 0.56 | 35.11 | 58.97 |
| Bayannaoer | 107.39 | 40.74 | 174 | 0.3 | 28.48 | 42.34 |
| Ulanqab | 113.13 | 40.99 | 272 | 0.19 | 14.49 | 24.08 |
| Wuhai | 106.8 | 39.65 | 44 | 0.61 | 34.91 | 70.16 |
| Jinzhong | 112.75 | 37.69 | 332 | 0.32 | 21.47 | 35.11 |
| Taiyuan | 112.56 | 37.88 | 369 | 0.47 | 58.2 | 99.09 |
| Jincheng | 112.85 | 35.49 | 221 | 0.38 | 25.93 | 37.97 |
| Datong | 113.3 | 40.08 | 318 | 0.45 | 29.76 | 48.91 |
| Changzhi | 113.12 | 36.2 | 338 | 0.34 | 24.41 | 41.77 |
| Xinzhou | 112.73 | 38.42 | 308 | 0.36 | 19.23 | 28.37 |
| Yuncheng | 111.01 | 35.03 | 513 | 0.52 | 22.58 | 43.69 |
| Shuozhou | 112.44 | 39.36 | 164 | 0.38 | 17.59 | 37 |
| Luliang | 111.14 | 37.52 | 392 | 0.27 | 18.37 | 21.97 |
| Yangquan | 113.58 | 37.86 | 132 | 0.37 | 29.49 | 44.03 |
| Linfen | 111.52 | 36.09 | 433 | 0.44 | 25.5 | 36.85 |
| Dalian | 121.61 | 38.91 | 595 | 0.26 | 34.12 | 69.65 |
| Huludao | 120.84 | 40.71 | 277 | 0.27 | 17.26 | 36.66 |
| Shenyang | 123.46 | 41.68 | 737 | 0.37 | 38.25 | 87.1 |
| Anshan | 122.99 | 41.11 | 344 | 0.3 | 19.42 | 55.45 |
| Jinzhou | 121.13 | 41.1 | 296 | 0.26 | 20.65 | 51.17 |
| Fuxin | 121.67 | 42.02 | 186 | 0.3 | 24.45 | 52.46 |
| Tieling | 123.84 | 42.29 | 294 | 0.52 | 19.39 | 43.42 |
| Chaoyang | 120.45 | 41.57 | 336 | 0.24 | 22.21 | 39.54 |
| Panjin | 122.07 | 41.12 | 130 | 0.48 | 31.22 | 69.38 |
| Dandong | 124.36 | 40 | 235 | 0.19 | 24.08 | 48.88 |
| Benxi | 123.77 | 41.29 | 148 | 0.28 | 26.8 | 72.87 |
| Liaoyang | 123.24 | 41.27 | 177 | 0.35 | 25.95 | 66.63 |
| Yingkou | 122.23 | 40.67 | 232 | 0.54 | 25.66 | 54.11 |
| Changchun | 125.32 | 43.82 | 749 | 0.22 | 28.2 | 59.75 |
| Liaoyuan | 125.14 | 42.89 | 118 | 0.18 | 23.8 | 39.85 |
| Siping | 124.35 | 43.17 | 320 | 0.2 | 22.43 | 40.25 |
| Tonghua | 125.94 | 41.73 | 217 | 0.3 | 27.68 | 46.17 |
| Baicheng | 122.84 | 45.62 | 191 | 0.21 | 24.54 | 32.97 |
| Jilin | 126.55 | 43.84 | 415 | 0.36 | 30.99 | 57.68 |
| Songyuan | 124.83 | 45.14 | 275 | 0.19 | 20.66 | 27.86 |
| Wuzhong | 106.2 | 38 | 143 | 0.35 | 18.51 | 40.44 |
| Yinchuan | 106.23 | 38.49 | 189 | 0.37 | 47.57 | 83.21 |
| Guyuan | 106.24 | 36.02 | 151 | 0.09 | 16.64 | 36.9 |
| Zhongwei | 105.2 | 37.5 | 122 | 0.23 | 15.3 | 32.73 |
| Shizuishan | 106.38 | 38.98 | 74 | 0.47 | 29.34 | 54.03 |
| Lanzhou | 103.83 | 36.06 | 326 | 0.39 | 42 | 77.86 |
| Pingliang | 106.67 | 35.54 | 234 | 0.64 | 19.63 | 53.38 |
| Dingxi | 104.63 | 35.58 | 303 | 0.16 | 15.79 | 39.19 |
| Baiyin | 104.14 | 36.54 | 182 | 0.56 | 18.36 | 42.49 |
| Qingyang | 107.64 | 35.71 | 270 | 0.1 | 15.37 | 27.89 |
| Tianshui | 105.72 | 34.58 | 371 | 0.13 | 13.6 | 29.03 |
| Longnan | 104.92 | 33.4 | 287 | 1.01 | 13.48 | 41.03 |
| Zhangye | 100.45 | 38.93 | 131 | 0.4 | 25.25 | 48.42 |
| Jinchang | 102.19 | 38.52 | 46 | 0.28 | 30.11 | 53.09 |
| Xining | 101.78 | 36.62 | 206 | 0.35 | 41.37 | 87.63 |
| Lhasa | 91.11 | 29.64 | 54 | 0.52 | 47.26 | 65.17 |
